# Supplementary material for: Development of a multi-locus CRISPR gene drive system in budding yeast
Source: Sci Rep. 2018 Nov 22;8:17277. doi: 10.1038/s41598-018-34909-3 (PMC6250742; doi:10.1038/s41598-018-34909-3)
Supplement: Supplementary file 1 — Supplemental Information [file 41598_2018_34909_MOESM1_ESM.docx]

**SUPPLEMENTARY INFORMATION**

For

**Development of a multi-locus CRISPR gene drive system in budding yeast**

Yao Yan^1^ and Gregory C. Finnigan^1*^

^1^Department of Biochemistry and Molecular Biophysics, Kansas State University, 141 Chalmers Hall, Manhattan, KS 66506 USA

*Correspondence to: Gregory C. Finnigan, Phone: (785) 532-6939; FAX; (785) 532-7278;

E-mail: [gfinnigan@ksu.edu](mailto:gfinnigan@ksu.edu)


**Figure S1.** Methodology for construction of a CRISPR gene drive system across three loci. This protocol is performed in haploid yeast. The final haploid strain harbors (inactive) Cas9 when grown on dextrose, and all three guide RNA cassettes. This method allows for a universal targeting strategy (Kan^R^ marker) and does not require gene-specific guides to be cloned or purchased. Moreover, integration of the guide cassette in place of the Kan^R^ marker allows for rapid screening of viable isolates following Cas9 editing (sensitivity to G418 indicates loss of the Kan^R^ cassette).

**Figure S2.** DNA sequences of engineered gene drives, guide RNA cassettes, and modified *DNL4* expression alleles used in this study.

**GFY-3675**

*prHIS3::****(u2)****::prGAL1/10::****SpCas9****::****NLS****::ADH1(t)::****(u2)****::HIS3(t)*

***(u2) 20 bp target and 3 bp PAM sequence***

**(992 bp 5’ UTR to *HIS3* shown)**

GGGTCAGTTATTTCATCCAGATATAACCCGAGAGGAAACTTCTTAGCGTCTGTTTTCGTACCATAAGGCAGTTCATGAGGTATATTTTCGTTATTGAAGCCCAGCTCGTGAATGCTTAATGCTGCTGAACTGGTGTCCATGTCGCCTAGGTACGCAATCTCCACAGGCTGCAAAGGTTTTGTCTCAAGAGCAATGTTATTGTGCACCCCGTAATTGGTCAACAAGTTTAATCTGTGCTTGTCCACCAGCTCTGTCGTAACCTTCAGTTCATCGACTATCTGAAGAAATTTACTAGGAATAGTGCCATGGTACAGCAACCGAGAATGGCAATTTCTACTCGGGTTCAGCAACGCTGCATAAACGCTGTTGGTGCCGTAGACATATTCGAAGATAGGATTATCATTCATAAGTTTCAGAGCAATGTCCTTATTCTGGAACTTGGATTTATGGCTCTTTTGGTTTAATTTCGCCTGATTCTTGATCTCCTTTAGCTTCTCGACGTGGGCCTTTTTCTTGCCATATGGATCCGCTGCACGGTCCTGTTCCCTAGCATGTACGTGAGCGTATTTCCTTTTAAACCACGACGCTTTGTCTTCATTCAACGTTTCCCATTGTTTTTTTCTACTATTGCTTTGCTGTGGGAAAAACTTATCGAAAGATGACGACTTTTTCTTAATTCTCGTTTTAAGAGCTTGGTGAGCGCTAGGAGTCACTGCCAGGTATCGTTTGAACACGGCATTAGTCAGGGAAGTCATAACACAGTCCTTTCCCGCAATTTTCTTTTTCTATTACTCTTGGCCTCCTCTAGTACACTCTATATTTTTTTATGCCTCGGTAATGATTTTCATTTTTTTTTTTCCACCTAGCGGATGACTCTTTTTTTTTCTTAGCGATTGGCATTATCACATAATGAATTATACATTATATAAAGTAATGTGATTTCTTCGAAGAATATACTAAAAAATGAGCAGGCAAGATAAACGAAGGCAAAG**GCTGTTCGTGTGCGCGTCCTGGG**GACAGGTTATCAGCAACAACACAGTCATATCCATTCTCAATTAGCTCTACCACAGTGTGTGAACCAATGTATCCAGCACCACCTGTAACCAAAACAATTTTAGAAGTACTTTCACTTTGTAACTGAGCTGTCATTTATATTGAATTTTCAAAAATTCTTACTTTTTTTTTGGATGGACGCAAAGAAGTTTAATAATCATATTACATGGCATTACCACCATATACATATCCATATACATATCCATATCTAATCTTACTTATATGTTGTGGAAATGTAAAGAGCCCCATTATCTTAGCCTAAAAAAACCTTCTCTTTGGAACTTTCAGTAATACGCTTAACTGCTCATTGCTATATTGAAGTACGGATTAGAAGCCGCCGAGCGGGTGACAGCCCTCCGAAGGAAGACTCTCCTCCGTGCGTCCTCGTCTTCACCGGTCGCGTTCCTGAAACGCAGATGTGCCTCGCGCCGCACTGCTCCGAACAATAAAGATTCTACAATACTAGCTTTTATGGTTATGAAGAGGAAAAATTGGCAGTAACCTGGCCCCACAAACCTTCAAATGAACGAATCAAATTAACAACCATAGGATGATAATGCGATTAGTTTTTTAGCCTTATTTCTGGGGTAATTAATCAGCGAAGCGATGATTTTTGATCTATTAACAGATATATAAATGCAAAAACTGCATAACCACTTTAACTAATACTTTCAACATTTTCGGTTTGTATTACTTCTTATTCAAATGTAATAAAAGTATCAACAAAAAATTGTTAATATACCTCTATACTTTAACGTCAAGGAGAAAAAACTATA**ATGGATAAGAAATACTCTATCGGTTTGGATATTGGTACAAATTCAGTTGGTTGGGCAGTTATTACTGATGAATACAAGGTTCCATCTAAAAAGTTTAAAGTTTTGGGTAACACTGATAGACATTCTATTAAGAAAAATTTGATTGGTGCTTTGTTATTTGATTCTGGTGAAACTGCTGAAGCAACAAGATTGAAAAGAACTGCAAGAAGAAGATACACAAGAAGAAAGAATAGAATCTGTTATTTGCAAGAAATTTTCTCTAACGAAATGGCTAAGGTTGATGATTCTTTCTTTCATAGATTGGAAGAATCATTTTTAGTTGAAGAAGATAAGAAACATGAAAGACATCCAATCTTCGGTAACATCGTTGATGAAGTTGCTTACCATGAAAAGTACCCAACAATCTATCATTTGAGAAAGAAATTGGTTGATTCAACTGATAAGGCAGATTTGAGATTGATATATTTGGCTTTAGCACATATGATCAAGTTTAGAGGTCATTTCTTGATCGAGGGTGACTTGAATCCAGATAATTCTGATGTTGATAAGTTGTTTATTCAATTAGTTCAAACATATAATCAATTGTTTGAAGAAAATCCAATTAATGCTTCTGGTGTTGATGCTAAGGCAATCTTGTCAGCAAGATTGTCTAAGTCAAGAAGATTGGAAAATTTGATCGCTCAATTACCAGGTGAAAAGAAAAATGGTTTGTTCGGTAATTTGATCGCATTGTCTTTGGGTTTGACACCAAACTTCAAGTCAAACTTCGATTTGGCTGAAGATGCAAAGTTGCAATTGTCTAAGGATACTTACGATGATGATTTGGATAATTTGTTGGCTCAAATTGGTGACCAATATGCAGATTTGTTTTTGGCTGCTAAAAATTTGTCTGATGCTATCTTGTTGTCAGATATCTTGAGAGTTAACACTGAAATCACAAAGGCTCCATTGTCTGCATCAATGATCAAGAGATACGATGAACATCATCAAGATTTGACTTTGTTGAAGGCATTGGTTAGACAACAATTACCAGAAAAGTACAAGGAAATTTTCTTTGATCAATCTAAAAATGGTTATGCTGGTTACATTGATGGTGGTGCATCTCAAGAAGAATTCTACAAGTTTATTAAGCCAATCTTGGAAAAGATGGATGGTACAGAAGAATTGTTAGTTAAATTGAACAGAGAAGATTTGTTAAGAAAACAAAGAACTTTCGATAACGGTTCTATCCCACATCAAATCCATTTGGGTGAATTACATGCTATCTTGAGAAGACAAGAAGATTTCTACCCATTTTTAAAGGATAACAGAGAAAAGATTGAAAAGATTTTGACTTTTAGAATTCCATATTACGTTGGTCCATTAGCTCGTGGTAATTCTAGATTTGCATGGATGACTAGAAAGTCAGAAGAAACTATCACACCATGGAATTTTGAAGAAGTTGTTGATAAAGGTGCTTCTGCACAATCTTTTATTGAAAGAATGACAAACTTCGATAAAAATTTGCCAAACGAAAAGGTTTTGCCAAAGCATTCATTGTTATATGAATACTTTACTGTTTACAATGAATTGACAAAAGTTAAATATGTTACTGAGGGTATGAGAAAACCAGCATTTTTGTCTGGTGAACAAAAGAAAGCAATCGTTGATTTGTTGTTTAAAACTAACAGAAAGGTTACAGTTAAACAATTGAAAGAAGATTACTTTAAGAAAATTGAATGTTTTGATTCTGTTGAAATTTCAGGTGTTGAAGATAGATTCAATGCTTCATTAGGTACTTACCATGATTTGTTGAAGATTATTAAGGATAAAGATTTCTTGGATAATGAAGAAAATGAAGATATTTTAGAAGATATTGTTTTAACTTTGACATTATTTGAAGATAGAGAAATGATCGAAGAAAGATTGAAGACATACGCTCATTTGTTCGATGATAAAGTTATGAAGCAATTGAAGAGAAGAAGATACACTGGTTGGGGTAGATTGTCTAGAAAGTTGATTAATGGTATCAGAGATAAGCAATCTGGTAAAACAATCTTGGATTTCTTGAAGTCAGATGGTTTCGCAAACAGAAACTTCATGCAATTGATTCATGATGATTCATTGACTTTTAAAGAAGATATCCAAAAAGCTCAAGTTTCTGGTCAGGGTGACTCATTGCATGAACATATTGCTAATTTGGCAGGTTCTCCAGCTATTAAGAAAGGTATCTTGCAAACAGTTAAGGTTGTTGATGAATTAGTTAAAGTTATGGGTAGACATAAGCCAGAAAACATCGTTATCGAAATGGCTAGAGAAAACCAAACTACACAAAAGGGTCAAAAGAATTCAAGAGAAAGAATGAAGAGAATCGAAGAAGGTATTAAAGAATTGGGTTCTCAAATCTTGAAGGAACATCCAGTTGAAAACACTCAATTGCAAAACGAAAAGTTGTACTTATACTACTTACAAAACGGTAGAGATATGTACGTTGATCAAGAATTAGATATCAACAGATTGTCAGATTACGATGTTGATCATATCGTTCCACAATCATTTTTGAAGGATGATTCAATCGATAATAAGGTTTTGACAAGATCTGATAAGAACCGTGGTAAATCTGATAATGTTCCATCAGAAGAAGTTGTTAAGAAAATGAAGAACTACTGGAGACAATTGTTAAATGCTAAGTTGATCACTCAAAGAAAGTTCGATAATTTGACAAAAGCTGAAAGAGGTGGTTTGTCAGAATTAGATAAAGCAGGTTTTATTAAGAGACAATTAGTTGAAACTAGACAAATCACAAAGCATGTTGCACAAATCTTGGATTCTAGAATGAACACTAAATATGATGAAAATGATAAATTAATTAGAGAAGTTAAAGTTATTACATTAAAATCTAAATTGGTTTCAGATTTTAGAAAAGATTTTCAATTCTACAAAGTTAGAGAAATTAATAACTATCATCATGCTCATGATGCATACTTGAATGCTGTTGTTGGTACTGCATTGATTAAGAAATACCCAAAGTTGGAATCTGAATTCGTTTACGGTGACTACAAGGTTTACGATGTTAGAAAGATGATCGCTAAGTCAGAACAAGAAATCGGTAAAGCTACAGCAAAGTATTTCTTTTATTCTAACATCATGAATTTCTTTAAAACTGAAATTACATTAGCTAACGGTGAAATCAGAAAAAGACCATTGATCGAAACTAATGGTGAAACAGGTGAAATTGTTTGGGATAAAGGTAGAGATTTCGCAACTGTTAGAAAGGTTTTGTCAATGCCACAAGTTAACATCGTTAAGAAAACTGAAGTTCAAACAGGTGGTTTTTCTAAGGAATCAATCTTGCCAAAGAGAAACTCTGATAAGTTGATTGCTAGAAAGAAAGATTGGGATCCAAAGAAATATGGTGGTTTTGATTCTCCAACTGTTGCTTACTCAGTTTTAGTTGTTGCAAAGGTTGAAAAGGGTAAATCTAAGAAATTGAAATCAGTTAAAGAATTGTTAGGTATCACAATCATGGAAAGATCTTCATTCGAAAAGAATCCAATCGATTTCTTGGAAGCAAAGGGTTACAAGGAAGTTAAGAAAGATTTGATTATTAAGTTGCCAAAGTACTCTTTGTTCGAATTAGAAAACGGTAGAAAAAGAATGTTAGCTTCAGCTGGTGAATTGCAAAAGGGTAATGAATTGGCTTTGCCATCTAAGTACGTTAATTTCTTGTATTTGGCATCTCATTACGAAAAGTTGAAGGGTTCACCAGAAGATAATGAACAAAAACAATTGTTCGTTGAACAACATAAGCATTATTTGGATGAAATTATTGAACAAATTTCTGAATTTTCAAAAAGAGTTATTTTGGCTGATGCAAATTTGGATAAGGTTTTGTCTGCTTACAATAAGCATAGAGATAAGCCAATCAGAGAACAAGCAGAAAACATCATCCATTTGTTTACTTTGACAAATTTGGGTGCTCCAGCTGCTTTTAAATACTTCGATACTACAATCGATAGAAAAAGATACACTTCTACAAAGGAAGTTTTGGATGCAACATTGATCCATCAATCAATCACTGGTTTGTATGAAACAAGAATTGATTTGTCTCAATTGGGTGGTGACTCTAGGGCAGACCCAAAGAAAAAGAGGAAAGTATAA**GGCGCGCCACTTCTAAATAAGCGAATTTCTTATGATTTATGATTTTTATTATTAAATAAGTTATAAAAAAAATAAGTGTATACAAATTTTAAAGTGACTCTTAGGTTTTAAAACGAAAATTCTTATTCTTGAGTAACTCTTTCCTGTAGGTCAGGTTGCTTTCTCAGGTATAGTATGAGGTCGCTCTTATTGACCACACCTCTACCGGCAGATCCGCTAGGGATAACAGGGTAATAT**GCTGTTCGTGTGCGCGTCCTGGG**TGACACCGATTATTTAAAGCTGCAGCATACGATATATATACATGTGTATATATGTATACCTATGAATGTCAGTAAGTATGTATACGAACAGTATGATACTGAAGATGACAAGGTAATGCATCATTCTATACGTGTCATTCTGAACGAGGCGCGCTTTCCTTTTTTCTTTTTGCTTTTTCTTTTTTTTTCTCTTGAACTCGAGAAAAAAAATATAAAAGAGATGGAGGAACGGGAAAAAGTTAGTTGTGGTGATAGGTGGCAAGTGGTATTCCGTAAGAACAACAAGAAAAGCATTTCATATTATGGCTGAACTGAGCGAACAAGTGCAAAATTTAAGCATCAACGACAACAACGAGAATGGTTATGTTCCTCCTCACTTAAGAGGAAAACCAAGAAGTGCCAGAAATAACAGTAGCAACTACAATAACAACAACGGCGGCTACAACGGTGGCCGTGGCGGTGGCAGCTTCTTTAGCAACAACCGTCGTGGTGGTTACGGCAACGGTGGTTTCTTCGGTGGAAACAACGGTGGCAGCAGATCTAACGGCCGTTCTGGTGGTAGATGGATCGATGGCAAACATGTCCCAGCTCCAAGAAACGAAAAGGCCGAGATCGCCATATTTGGTGTCCCCGAGGATCCAAATTTCCAATCTTCTGGTATTAACTTCGATAACTACGATGATATTCCAGTGGACGCCTCTGGTAAGGATGTTCCTGAACCAATCACAGAATTTACCTCACCTCCATTGGACGGATTGTTATTGGAAAACATCAAATTGGCCCGTTTCACCAAGCCAACACCTGTGCAAAAATACTCCGTCCCTATCGTTGCCAACGGCAGAGATTTGATGGCCTGTGCGCAGACCGGTTCTGGTAAGACTGGTGGGTTTTTATTCCCAGTGTTGTCCGAATCATTTAAGACTGGACCATCTCCTCAACCAGAGTCTCAAGGCTCCTTTTACCAAAGAAAGGCCTACCCAACTGCTGTCATTA

**(993 bp 3’ UTR to *HIS3* shown)**

***prSHS1::sgRNA(GFP)::SHS1(t)***

*prSNR52****::crRNA::****tracrRNA****::SUP4(t)***

**(596 bp 5’ UTR to *SHS1* shown)**

GGTTATCGTATTTCACTTTTTGTGGTAAACTCAGGTCAATATTATGCGACTTGAACCATTCAGTCAAAGGTTCGTTACCACCTTTTTCCATACGAAGAAGTTCCTCCGGTTTAAACTGATCCATAGTGATAGATCTTACAAAAGATATATGCACACCAAGCCCTCTATGGATACCGGCACATTCAAGGCAAATGAAAGCTCCAAACTTAGGCGTGGCCCATTGTGGATTTGGCGCACCACAATCCATACATTTCTTATTTGCACCAATCTTTTGCAATTGCAAAAGACGCCTGCGGGTATCTGGGTCCACTTTCCAATCTGACATGCTCTATAATCCGCGATAAAATTGCTCAATTGGCACCATTTAAACTCAGAATCACGTCCATATTTCTGCTTTCATTCTTGATATAGTTGTGCAATTTGGTTCTTGACAAAAACTGGCGTTGCTTGCGGGTAACCGCGCGATTTTTAAAGTGCCAAACTGCGAAAAGAATATAACAAGCTTTCGAGCAAGATCAATGTACCCAGCAAGTGAAATAATAAAACAAGAGCCCCAAAGATCTGCTTATAATTGCTAGAAAAATATATTATTAATC**GGATCC**TCACTAA**AGGGAACAAAAGCTGGAGCT**TCTTTGAAAAGATAATGTATGATTATGCTTTCACTCATATTTATACAGAAACTTGATGTTTTCTTTCGAGTATATACAAGGTGATTACATGTACGTTTGAAGTACAACTCTAGATTTTGTAGTGCCCTCTTGGGCTAGCGGTAAAGGTGCGCATTTTTTCACACCCTACAATGTTCTGTTCAAAAGATTTTGGTCAAACGCTGTAGAAGTGAAAGTTGGTGCGCATGTTTCGGCGTTCGAAACTTCTCCGCAGTGAAAGATAAATGATC**AAAGGAGAAGAACTTTTCAC**GTTTTAGAGCTAGAAATAGCAAGTTAAAATAAGGCTAGTCCGTTATCAACTTGAAAAAGTGGCACCGAGTCGGTGGTGC**TTTTTTTGTTTTTTATGTCTTCGAGTCATGTAATTAGTTA**TGTCACGC**CTCGAG**GTTGTATCTGTACAAAATCCAAAGCTGAGCAAATAAATAAATAAATAAATGTATAAGTTACCGAACGGGGGTATTTTTACTTTTGATCAAAAATTTATGTACCAACTACAAAGTTTCCTCAGCACAGCCTTCAAGAAGGGAACACACATACAAACAGTGTCAAATAATTGTAGGGATAAATTTAAATATGGCATAAACTAAATAAGTAGAGCATGAAAAAACTGCAAAATCCAAAAAGTAAAAACGAAGGTCAGAAAGTAAAGCAAAAGAAAATTAATAAAGCAATACTAAATCTATCATGATTTCCCGTAACTTCCATTAAAGCTGTAACCAGATTTACTCCTACTGTTTGAGCCTCTAACGCCTAATGGATTTTTAGAGAAGCTCAACCTGATACCTCCTTTGTTGTTGAGGGAAGGGCGGGGGTGAGGTAGTTGACTACCATATAATTCTGCCAATGCTCTAGTGGCAAAGCTAACATCCTC

**(485 bp 3’ UTR to *SHS1* shown)**

***prDNL4::sgRNA(Kan)::DNL4(t)***

*prSNR52****::crRNA::****tracrRNA****::SUP4(t)***

**(590 bp 5’ UTR to *DNL4* shown)**

GGATGATGGGTAACATAAATAGAAGGGGTAATGGTTCGCAATCTGACACATCAGAGAGTGAGGAAAACTCAGAACAATCTGATTTGGAAGGCAATAATCAATGTATTGAATATGACTCTTTAGGTAATGCTATTCGTATAGATAACATGAAAAGCAGGGAAGCGCAATCTGAGGAATCAGAAGACGAGGAAAGTGGTTCAAAAGAAAATGGAGAGCCTTTAAGTTATGACCCCTTAGGCAATTTAATTCGATAGGTGATTAAATAGGCTGAAATCAGTGTTTAGTAACTACGTACGTTGTACATGTAACATTGTGATATAAATCGTAAGATTCGCCGAGTATAGATCAATAATATCGGTTTCATCACTTACGTTGTTTGTGCAGTACTAGAGTTAAGATCGTTTTCGATCCCTTATTTTCTTCTTTTTTCCTTTTTTTTGTTATTTTTCTCTTTTTACCTTTTGTCACCATATTAAATCTTTAAACAAATCTAACTATGAAAAAATCCTTTAAACATATGTTAATATGTGGAAAATAAATACTAAAATAAAAATCTAGAACTGAAGGAAATAGTAACGGATTATTTAGGT**GGATCC**TCACTAAAGGGAACAAAAGCTGGAGCTTCTTTGAAAAGATAATGTATGATTATGCTTTCACTCATATTTATACAGAAACTTGATGTTTTCTTTCGAGTATATACAAGGTGATTACATGTACGTTTGAAGTACAACTCTAGATTTTGTAGTGCCCTCTTGGGCTAGCGGTAAAGGTGCGCATTTTTTCACACCCTACAATGTTCTGTTCAAAAGATTTTGGTCAAACGCTGTAGAAGTGAAAGTTGGTGCGCATGTTTCGGCGTTCGAAACTTCTCCGCAGTGAAAGATAAATGATC**GCCATCCTATGGAACTGCCT**GTTTTAGAGCTAGAAATAGCAAGTTAAAATAAGGCTAGTCCGTTATCAACTTGAAAAAGTGGCACCGAGTCGGTGGTGC**TTTTTTTGTTTTTTATGTCT**TCGAGTCATGTAATTAGTTATGTCACGC**CTCGAG**TGGTGCGTTTTGCGGAGGCTTAATTTTTTGAAGTTTATTTAATACTATCCTACATATGTACATTAAATACTTCCGTAACGTTTATCAATAAGAGTGGAAGATGCGCAATTATATTCAAAAGATTGGCCAGTCAATTAACTTAAGGAAAAAATTTACTGCGCGGCTGTGGTCCCATTTGAAACGGGGACGTTAGTGTTGCTTTTGTGTTTCCTTCTTCTTCTTTTTTTCTTTTCTTTAGAAGTATCTTCTTTGTCTTTCACCTCATCGCTGGGCGAGTTGACTTTCTTCTTCTTCTTTACGCTCAAAGGATTAGGAGCCTTTGGACCAAGCTTTCTTTTCTTAGTGATAGATTCCTTTCCTGATCCATCACCACTTTCTTGAAGTTTTTCAATATTAGGATCATTGAGACCCTTATACAATTTTTGTTCTTCAGTAATCTTGCTTGCCTTGGCACTCGCCGTACTGAGAGGTTCCATAACCATAACAGACCTGGTTAAATGGATTAATGGAACACCCGGAACCGTCCTCAGCTTCCTCCTCAAATCTATGTCCTGAGAAGCAACCACATACCTGTGTTTATTTGCACCGC

**(589 bp 3’ UTR to *DNL4* shown)**

**GFY-3596**

*prHIS3****::(u1)::****prCDC12****::mCherry::NLS::SHS1(t)::****prCCW12****::SpHIS5::****MX(t)::(****u1)::****HIS3(t)*

***(u1) 20 bp target and 3 bp PAM sequence***

**(992 bp 5’ UTR to *HIS3* shown)**

GGGTCAGTTATTTCATCCAGATATAACCCGAGAGGAAACTTCTTAGCGTCTGTTTTCGTACCATAAGGCAGTTCATGAGGTATATTTTCGTTATTGAAGCCCAGCTCGTGAATGCTTAATGCTGCTGAACTGGTGTCCATGTCGCCTAGGTACGCAATCTCCACAGGCTGCAAAGGTTTTGTCTCAAGAGCAATGTTATTGTGCACCCCGTAATTGGTCAACAAGTTTAATCTGTGCTTGTCCACCAGCTCTGTCGTAACCTTCAGTTCATCGACTATCTGAAGAAATTTACTAGGAATAGTGCCATGGTACAGCAACCGAGAATGGCAATTTCTACTCGGGTTCAGCAACGCTGCATAAACGCTGTTGGTGCCGTAGACATATTCGAAGATAGGATTATCATTCATAAGTTTCAGAGCAATGTCCTTATTCTGGAACTTGGATTTATGGCTCTTTTGGTTTAATTTCGCCTGATTCTTGATCTCCTTTAGCTTCTCGACGTGGGCCTTTTTCTTGCCATATGGATCCGCTGCACGGTCCTGTTCCCTAGCATGTACGTGAGCGTATTTCCTTTTAAACCACGACGCTTTGTCTTCATTCAACGTTTCCCATTGTTTTTTTCTACTATTGCTTTGCTGTGGGAAAAACTTATCGAAAGATGACGACTTTTTCTTAATTCTCGTTTTAAGAGCTTGGTGAGCGCTAGGAGTCACTGCCAGGTATCGTTTGAACACGGCATTAGTCAGGGAAGTCATAACACAGTCCTTTCCCGCAATTTTCTTTTTCTATTACTCTTGGCCTCCTCTAGTACACTCTATATTTTTTTATGCCTCGGTAATGATTTTCATTTTTTTTTTTCCACCTAGCGGATGACTCTTTTTTTTTCTTAGCGATTGGCATTATCACATAATGAATTATACATTATATAAAGTAATGTGATTTCTTCGAAGAATATACTAAAAAATGAGCAGGCAAGATAAACGAAGGCAAAG**ATGACGGTGGACTTCGGCTACGTAGGGCGATT**GGGGCAGCGCCCTGTTTTTCATTAATGTAGTCAGCAATGTCAAGATTCAACGCCAAGTCTGGTTCAGCAAGTGACATTCTGCAAGCTCTTTGAATCTTCCTCAAAAGAGGATTGCCCAAGGCTTGAGGTTTCCTGACGGGCAACTCAGACAAATATATGCTATGTGAGTGCGGATGGGACATGATGCAGTATCACGATTAGCAATTCAGCTATGAGTTATGTTGCTCTTTGTTTTGTTTATGGAAATTGTCCTATGGTAAGTCTCTTTTTTTTGCAATCGTGATTACAGAAAAAAAACAGGGCGCTGGAAAAGTGAAGAATCCGAAATTTTTTTCGAAATCACCATTGTTTGTTTTGAGTAGATCAAAGTCTTGAAAGGTGCAGCAAGATATAGGATCTTGACCTGAAGAGTATTGATAACGAACTACATCACATATTGTATCAAATA**ATGGTGAGCAAGGGCGAGGAGGATAACATGGCCATCATCAAGGAGTTCATGCGCTTCAAGGTGCACATGGAGGGCTCCGTGAACGGCCACGAGTTCGAGATCGAGGGCGAGGGCGAGGGCCGCCCCTACGAGGGCACCCAGACCGCCAAGCTGAAGGTGACCAAGGGTGGCCCCCTGCCCTTCGCCTGGGACATCCTGTCCCCTCAGTTCATGTACGGCTCCAAGGCCTACGTGAAGCACCCCGCCGACATCCCCGACTACTTGAAGCTGTCCTTCCCCGAGGGCTTCAAGTGGGAGCGCGTGATGAACTTCGAGGACGGCGGCGTGGTGACCGTGACCCAGGACTCCTCCCTGCAGGACGGCGAGTTCATCTACAAGGTGAAGCTGCGCGGCACCAACTTCCCCTCCGACGGCCCCGTAATGCAGAAGAAGACCATGGGCTGGGAGGCCTCCTCCGAGCGGATGTACCCCGAGGACGGCGCCCTGAAGGGCGAGATCAAGCAGAGGCTGAAGCTGAAGGACGGCGGCCACTACGACGCTGAGGTCAAGACCACCTACAAGGCCAAGAAGCCCGTGCAGCTGCCCGGCGCCTACAACGTCAACATCAAGTTGGACATCACCTCCCACAACGAGGACTACACCATCGTGGAACAGTACGAACGCGCCGAGGGCCGCCACTCCACCGGCGGCATGGACGAGCTGTACAAGTCTAGGGCAGACCCAAAGAAAAAGAGGAAAGTATAA**GTTGTATCTGTACAAAATCCAAAGCTGAGCAAATAAATAAATAAATAAATGTATAAGTTACCGAACGGGGGTATTTTTACTTTTGATCAAAAATTTATGTACCAACTACAAAGTTTCCTCAGCACAGCCTTCAAGAAGGGAACACACATACAAACAGTGTCAAATAATTGTAGGGATAAATTTAAATATGGCATAAACTAAATAAGTAGAGCATGAAAAAACTGCAAAATCCAAAAAGTAAAAACGAAGGTCAGAAAGTAAAGCAAAAGAAAATTAATAAAGCAATACTAAATCTATCATGATTTCCCGTAACTTCCATTAAAGCTGTAACCAGATTTACTCCTACTGTTTGAGCCTCTAACGCCTAATGGATTTTTAGAGAAGCTCAACCTGATACCTCCTTTGTTGTTGAGGGAAGGGCGGGGGTGAGGTAGTTGACTACCATATAATTCTGCCAATGCTCTAGTGGCAAAGCTAACATCCTCACAAAGCAAAATAAAAGAAACTTAATACGTTATGCCGTAATGAAGGGCTACCAAAAACGATAATCTCAACTGTAAACAGGTACAATGCGGACCCTTTTGCCACAAAACATACATCATTCATTGCCGGAAAAAGAAAGAAGTGAAGACAGCAGTGCAGCCAGCCATGTTGCGCCAATCTAATTATAGATGCTGGTGCCCTGAGGATGTATCTGGAGCCAGCCATGGCATCATGCGCTACCGCCGGATGTAAAATCCGACACGCAAAAGAAAACCTTCGAGGTTGCGCACTTCGCCCACCCATGAACCACACGGTTAGTCCAAAAGGGGCAGTTCAGATTCCAGATGCGGGAATTAGCTTGCTGCCACCCTCACCTCACTAACGCTGCGGTGTGCGGATACTTCATGCTATTTATAGACGCGCGTGTCGGAATCAGCACGCGCAAGAACCAAATGGGAAAATCGGAATGGGTCCAGAACTGCTTTGAGTGCTGGCTATTGGCGTCTGATTTCCGTTTTGGGAATCCTTTGCCGCGCGCCCCTCTCAAAACTCCGCACAAGTCCCAGAAAGCGGGAAAGAAATAAAACGCCACCAAAAAAAAAAAAATAAAAGCCAATCCTCGAAGCGTGGGTGGTAGGCCCTGGATTATCCCGTACAAGTATTTCTCAGGAGTAAAAAAACCGTTTGTTTTGGAATTCCCCATTTCGCGGCCACCTACGCCGCTATCTTTGCAACAACTATCTGCGATAACTCAGCAAATTTTGCATATTCGTGTTGCAGTATTGCGATAATGGGAGTCTTACTTCCAACATAACGGCAGAAAGAAATGTGAGAAAATTTTGCATCCTTTGCCTCCGTTCAAGTATATAAAGTCGGCATGCTTGATAATCTTTCTTTCCATCCTACATTGTTCTAATTATTCTTATTCTCCTTTATTCTTTCCTAACATACCAAGAAATTAATCTTCTGTCATTCGCTTAAACACTATATCAATA**ATGAGGAGGGCTTTTGTAGAAAGAAATACGAACGAAACGAAAATCAGCGTTGCCATCGCTTTGGACAAAGCTCCCTTACCTGAAGAGTCGAATTTTATTGATGAACTTATAACTTCCAAGCATACAAACCAAAAGGGAGAACAAGTAATCCAAGTAGACACGGGAATTGGATTCTTGGATCACATGTATCATGCACTGGCTAAACATGCAGGCTGGAGCTTACGACTTTACTCAAGAGGTGATTTAATCATCGATGATCATCACACTGCAGAAGATACTGCTATTGCACTTGGTATTGCATTCAAGCAGGCTATGAGTAACTTTGCCGGCGTTAAAAGATTTGGACATGCTTATTGTCCACTTGACGAAGCTCTTTCTAGAAGCGTAGTTGACTTGTCGGGACGGCCCTATGCTGTTATCGATTTGGGATTAAAGCGTGAAAAGGTTGGGGAATTGTCCTGTGAAATGATCCCTCACTTACTATATTCCTTTTCGGTAGCAGCTGGAATTACTTTGCATGTTACCTGCTTATATGGTAGTAATGACCATCATCGTGCTGAAAGCGCTTTTAAATCTCTGGCTGTTGCCATGCGCGCGGCTACTAGTCTTACTGGAAGTTCTGAAGTCCCAAGCACGAAGGGAGTGTTGTAA**AGAGTACTGACAATAAAAAGATTCTTGTTTTCAAGAACTTGTCATTTGTATAGTTTTTTTATATTGTAGTTGTTCTATTTTAATCAAATGTTAGCGTGATTTATATTTTTTTTCGCCTCGACATCATCTGCCCAGATGCGAAGTTAAGTGCGCAGAAAGTAATATCATGCGTCAATCGTATGTGAATGCTGGTCGCTATACTGCTGTCGATTCGATACTAACGCCGCCATCCAGT**ATGACGGTGGACTTCGGCTACGTAGGGCGATT**TGACACCGATTATTTAAAGCTGCAGCATACGATATATATACATGTGTATATATGTATACCTATGAATGTCAGTAAGTATGTATACGAACAGTATGATACTGAAGATGACAAGGTAATGCATCATTCTATACGTGTCATTCTGAACGAGGCGCGCTTTCCTTTTTTCTTTTTGCTTTTTCTTTTTTTTTCTCTTGAACTCGAGAAAAAAAATATAAAAGAGATGGAGGAACGGGAAAAAGTTAGTTGTGGTGATAGGTGGCAAGTGGTATTCCGTAAGAACAACAAGAAAAGCATTTCATATTATGGCTGAACTGAGCGAACAAGTGCAAAATTTAAGCATCAACGACAACAACGAGAATGGTTATGTTCCTCCTCACTTAAGAGGAAAACCAAGAAGTGCCAGAAATAACAGTAGCAACTACAATAACAACAACGGCGGCTACAACGGTGGCCGTGGCGGTGGCAGCTTCTTTAGCAACAACCGTCGTGGTGGTTACGGCAACGGTGGTTTCTTCGGTGGAAACAACGGTGGCAGCAGATCTAACGGCCGTTCTGGTGGTAGATGGATCGATGGCAAACATGTCCCAGCTCCAAGAAACGAAAAGGCCGAGATCGCCATATTTGGTGTCCCCGAGGATCCAAATTTCCAATCTTCTGGTATTAACTTCGATAACTACGATGATATTCCAGTGGACGCCTCTGGTAAGGATGTTCCTGAACCAATCACAGAATTTACCTCACCTCCATTGGACGGATTGTTATTGGAAAACATCAAATTGGCCCGTTTCACCAAGCCAACACCTGTGCAAAAATACTCCGTCCCTATCGTTGCCAACGGCAGAGATTTGATGGCCTGTGCGCAGACCGGTTCTGGTAAGACTGGTGGGTTTTTATTCCCAGTGTTGTCCGAATCATTTAAGACTGGACCATCTCCTCAACCAGAGTCTCAAGGCTCCTTTTACCAAAGAAAGGCCTACCCAACTGCTGTC

**(993 bp 3’ UTR to *HIS3* shown)**

*prSHS1****::SHS1::GFP::CDC10(t)::****prMX****::CaURA3::****SHS1(t)*

**(596 bp 5’ UTR to *SHS1* shown), (Gly polymorphism at codon 314)**

GGTTATCGTATTTCACTTTTTGTGGTAAACTCAGGTCAATATTATGCGACTTGAACCATTCAGTCAAAGGTTCGTTACCACCTTTTTCCATACGAAGAAGTTCCTCCGGTTTAAACTGATCCATAGTGATAGATCTTACAAAAGATATATGCACACCAAGCCCTCTATGGATACCGGCACATTCAAGGCAAATGAAAGCTCCAAACTTAGGCGTGGCCCATTGTGGATTTGGCGCACCACAATCCATACATTTCTTATTTGCACCAATCTTTTGCAATTGCAAAAGACGCCTGCGGGTATCTGGGTCCACTTTCCAATCTGACATGCTCTATAATCCGCGATAAAATTGCTCAATTGGCACCATTTAAACTCAGAATCACGTCCATATTTCTGCTTTCATTCTTGATATAGTTGTGCAATTTGGTTCTTGACAAAAACTGGCGTTGCTTGCGGGTAACCGCGCGATTTTTAAAGTGCCAAACTGCGAAAAGAATATAACAAGCTTTCGAGCAAGATCAATGTACCCAGCAAGTGAAATAATAAAACAAGAGCCCCAAAGATCTGCTTATAATTGCTAGAAAAATATATTATTAATC**ATGAGCACTGCTTCAACACCGCCAATTAACTTATTTCGTAGAAAGAAAGAACATAAACGTGGGATCACATACACAATGTTACTATGTGGGCCAGCAGGTACAGGAAAGACCGCCTTTGCTAACAATCTATTGGAAACTAAGATCTTTCCGCATAAGTATCAATACGGTAAATCAAATGCTAGTATTAGCTCTAACCCAGAAGTAAAAGTTATTGCTCCGACAAAAGTTGTTTCATTTAATTCGAAAAATGGGATTCCATCTTATGTTTCTGAATTCGATCCAATGAGAGCCAATTTGGAACCAGGTATTACCATCACCTCCACTTCATTAGAACTTGGGGGCAACAAAGATCAAGGAAAGCCAGAAATGAACGAGGATGATACCGTGTTTTTCAACTTGATTATGACGCATGGTATAGGCGAAAACTTGGACGATTCGTTGTGTTCTGAGGAAGTTATGTCGTATTTAGAACAACAATTTGACATTGTTTTAGCTGAGGAAACCAGAATTAAAAGGAATCCGAGGTTTGAGGACACCAGGGTTCACGTAGCATTATATTTTATTGAACCCACTGGACACGGTCTGAGAGAAGTCGATGTAGAGCTCATGAAAAGCATCTCCAAATACACAAATGTACTGCCAATAATAACAAGAGCTGACTCATTCACCAAGGAGGAGCTAACTCAATTCAGGAAAAATATTATGTTTGATGTGGAAAGATACAACGTCCCAATTTACAAATTTGAGGTTGACCCTGAAGATGATGATTTGGAATCCATGGAAGAGAATCAAGCCTTGGCATCCTTGCAACCATTTGCTATTATAACTTCAGATACCAGAGATAGTGAAGGTAGATACGTTAGGGAGTATCCGTGGGGGATAATATCAATCGACGACGACAAAATTTCGGATTTGAAAGTTTTAAAAAACGTCCTGTTTGGTTCTCACTTACAAGAATTCAAAGACACCACGCAAAATTTGCTTTACGAGAATTACCGTTCCGAAAAACTATCGTCCGTGGCCAACGCTGAAGAAATTGGTCCTAATTCTACAAAGAGACAGTCAAATGCTCCAAGTTTAAGCAACTTTGCCTCTTTGATAAGCACTGGTCAATTCAATTCTTCTCAAACTCTTGCAAACAATTTGAGAGCGGACACACCAAGAAACCAAGTAAGTGGAAACTTTAAGGAAAACGAATACGAAGACAATGGCGAACATGATTCAGCAGAAAATGAACAGGAAATGTCTCCCGTGAGACAGTTGGGTAGAGAAATAAAACAAGAAAATGAAAATTTGATAAGATCTATCAAAACAGAATCTTCACCAAAATTCTTGAACTCTCCGGACTTACCAGAGCGTACCAAGTTAAGAAATATTTCAGAAACCGTTCCATATGTCTTGAGACATGAAAGAATTTTAGCAAGACAACAAAAACTGGAAGAGTTAGAGGCCCAGTCAGCTAAAGAATTACAAAAAAGAATTCAAGAATTAGAAAGAAAAGCACACGAATTGAAATTGAGGGAAAAACTAATAAATCAGAATAAACTAAACGGTTCATCATCTTCAATCAATTCTCTACAACAGAGCACAAGGAGCCAAATTAAAAAAAATGACACGTATACTGATTTAGCCTCTATTGCATCGGGTAGAGATGGTCGACGGATCCCCGGGTTAATTAACAGTAAAGGAGAAGAACTTTTCACTGGAGTTGTCCCAATTCTTGTTGAATTAGATGGTGATGTTAATGGGCACAAATTTTCTGTCAGTGGGGAGGGTGAAGGTGATGCAACATACGGAAAACTTACCCTTAAATTTATTTGCACTACTGGAAAACTACCTGTTCCATGGCCAACACTTGTCACTACTTTGACTTATGGTGTTCAATGCTTTTCAAGATACCCAGATCATATGAAACGGCATGACTTTTTCAAGAGTGCCATGCCCGAAGGTTATGTACAGGAAAGAACTATATTTTTCAAAGATGACGGGAACTACAAGACACGTGCTGAAGTCAAGTTTGAAGGTGATACCCTTGTTAATAGAATCGAGTTAAAAGGTATTGATTTTAAAGAAGATGGAAACATTCTTGGACACAAATTGGAATACAACTATAACTCACACAATGTATACATCATGGCAGACAAACAAAAGAATGGAATCAAAGTTAACTTCAAAATTAGACACAACATTGAAGATGGAAGCGTTCAACTAGCAGACCATTATCAACAAAATACTCCAATTGGCGATGGCCCTGTCCTTTTACCAGACAACCATTACCTGTCCACACAATCTGCCCTTTCGAAAGATCCCAACGAAAAGAGAGACCACATGGTCCTTCTTGAGTTTGTAACAGCTGCTGGGATTACACATGGCATGGATGAACTATACAAATAG**ATCTCATAAGAATGGTGGTGATTATATATCTTATGTTATTAAGAATTCTCAAATTATTCTATATGAAAACACCGTAACTTGCTTCTCTCCTTGGTTTTACATAATGACATAATGCGATCGAAAACTAGAGGTACAGGTATTGCTGGATTGGCGAGAGTTTTTACCTTCTTTTCTGGCGTACAGCTATCACCTTCTCGTTTGGTAAAATGAAAGAACATTTTGTTGTCTTAGCCAAATATTTAATCTATGAAGAAAACGGAGTTTACCGGTAATTCTAAATAAAAGTTTGGTTAGGGATTGTGCCTCATAGAGAAGCAATTGGTACTCATCTTATTAAAGTATTACTATAAACATTAGAAAGAGTCCCTGAGCGTTGCTAATGGGAAGCTATTCGCGCTTTTAGTAAATTATTAAAATACGCCAAAAATAAATGTAATCCGGATATACCTCTTCTTTTAACCTTCCCAGATCTGTTTAGCTTGCCTCGTCCCCGCCGGGTCACCCGGCCAGCGACATGGAGGCCCAGAATACCCTCCTTGACAGTCTTGACGTGCGCAGCTCAGGGGCATGATGTGACTGTCGCCCGTACATTTAGCCCATACATCCCCATGTATAATCATTTGCATCCATACATTTTGATGGCCGCACGGCGCGAAGCAAAAATTACGGCTCCTCGCTGCAGACCTGCGAGCAGGGAAACGCTCCCCTCACAGACGCGTTGAATTGTCCCCACGCCGCGCCCCTGTAGAGAAATATAAAAGGTTAGGATTTGCCACTGAGGTTCTTCTTTCATATACTTCCTTTTAAAATCTTGCTAGGATACAGTTCTCACATCACATCCGAACATAAACAACC**ATGACAGTCAACACTAAGACCTATAGTGAGAGAGCAGAAACTCATGCCTCACCAGTAGCACAACGATTATTTCGATTAATGGAACTGAAGAAAACCAATTTATGTGCATCAATTGATGTTGATACCACTAAGGAATTCCTTGAATTAATTGATAAATTGGGTCCTTATGTATGCTTAATCAAGACTCATATTGATATAATCAATGATTTTTCCTATGAATCCACTATTGAACCATTATTAGAACTTTCACGTAAACATCAATTTATGATTTTTGAAGATAGAAAATTTGCTGATATTGGTAATACCGTGAAGAAACAATATATTGGTGGAGTTTATAAAATTAGTAGTTGGGCAGATATTACTAATGCTCATGGTGTCACTGGGAATGGAGTAGTTGAAGGATTAAAACAGGGAGCTAAAGAAACCACCACCAACCAAGAGCCAAGAGGGTTATTGATGTTAGCTGAATTATCATCAGTGGGATCATTAGCATATGGAGAATATTCTCAAAAAACTGTTGAAATTGCTAAATCCGATAAGGAATTTGTTATTGGATTTATTGCCCAACGTGATATGGGTGGACAAGAAGAAGGATTTGATTGGCTTATTATGACACCTGGAGTTGGATTAGATGATAAAGGTGATGGATTAGGACAACAATATAGAACTGTTGATGAAGTTGTTAGCACTGGAACTGATATTATCATTGTTGGTAGAGGATTGTTTGGTAAAGGAAGAGATCCAGATATTGAAGGTAAAAGGTATAGAGATGCTGGTTGGAATGCTTATTTGAAAAAGACTGGCCAATTATAA**GTTGTATCTGTACAAAATCCAAAGCTGAGCAAATAAATAAATAAATAAATGTATAAGTTACCGAACGGGGGTATTTTTACTTTTGATCAAAAATTTATGTACCAACTACAAAGTTTCCTCAGCACAGCCTTCAAGAAGGGAACACACATACAAACAGTGTCAAATAATTGTAGGGATAAATTTAAATATGGCATAAACTAAATAAGTAGAGCATGAAAAAACTGCAAAATCCAAAAAGTAAAAACGAAGGTCAGAAAGTAAAGCAAAAGAAAATTAATAAAGCAATACTAAATCTATCATGATTTCCCGTAACTTCCATTAAAGCTGTAACCAGATTTACTCCTACTGTTTGAGCCTCTAACGCCTAATGGATTTTTAGAGAAGCTCAACCTGATACCTCCTTTGTTGTTGAGGGAAGGGCGGGGGTGAGGTAGTTGACTACCATATAATTCTGCCAATGCTCTAGTGGCAAAGCTAACATCCTC

**(485 bp 3’ UTR to *SHS1* shown)**

*prDNL4****::prMX::Kan^R^::MX(t)::****DNL4(t)*

**(590 bp 5’ UTR to *DNL4* shown)**

GGATGATGGGTAACATAAATAGAAGGGGTAATGGTTCGCAATCTGACACATCAGAGAGTGAGGAAAACTCAGAACAATCTGATTTGGAAGGCAATAATCAATGTATTGAATATGACTCTTTAGGTAATGCTATTCGTATAGATAACATGAAAAGCAGGGAAGCGCAATCTGAGGAATCAGAAGACGAGGAAAGTGGTTCAAAAGAAAATGGAGAGCCTTTAAGTTATGACCCCTTAGGCAATTTAATTCGATAGGTGATTAAATAGGCTGAAATCAGTGTTTAGTAACTACGTACGTTGTACATGTAACATTGTGATATAAATCGTAAGATTCGCCGAGTATAGATCAATAATATCGGTTTCATCACTTACGTTGTTTGTGCAGTACTAGAGTTAAGATCGTTTTCGATCCCTTATTTTCTTCTTTTTTCCTTTTTTTTGTTATTTTTCTCTTTTTACCTTTTGTCACCATATTAAATCTTTAAACAAATCTAACTATGAAAAAATCCTTTAAACATATGTTAATATGTGGAAAATAAATACTAAAATAAAAATCTAGAACTGAAGGAAATAGTAACGGATTATTTAGGTGTTTAGCTTGCCTCGTCCCCGCCGGGTCACCCGGCCAGCGACATGGAGGCCCAGAATACCCTCCTTGACAGTCTTGACGTGCGCAGCTCAGGGGCATGATGTGACTGTCGCCCGTACATTTAGCCCATACATCCCCATGTATAATCATTTGCATCCATACATTTTGATGGCCGCACGGCGCGAAGCAAAAATTACGGCTCCTCGCTGCAGACCTGCGAGCAGGGAAACGCTCCCCTCACAGACGCGTTGAATTGTCCCCACGCCGCGCCCCTGTAGAGAAATATAAAAGGTTAGGATTTGCCACTGAGGTTCTTCTTTCATATACTTCCTTTTAAAATCTTGCTAGGATACAGTTCTCACATCACATCCGAACATAAACAACC**ATGGGTAAGGAAAAGACTCACGTTTCGAGGCCGCGATTAAATTCCAACATGGATGCTGATTTATATGGGTATAAATGGGCTCGCGATAATGTCGGGCAATCAGGTGCGACAATCTATCGATTGTATGGGAAGCCCGATGCGCCAGAGTTGTTTCTGAAACATGGCAAAGGTAGCGTTGCCAATGATGTTACAGATGAGATGGTCAGACTAAACTGGCTGACGGAATTTATGCCTCTTCCGACCATCAAGCATTTTATCCGTACTCCTGATGATGCATGGTTACTCACCACTGCGATCCCCGGCAAAACAGCATTCCAGGTATTAGAAGAATATCCTGATTCAGGTGAAAATATTGTTGATGCGCTGGCAGTGTTCCTGCGCCGGTTGCATTCGATTCCTGTTTGTAATTGTCCTTTTAACAGCGATCGCGTATTTCGTCTCGCTCAGGCGCAATCACGAATGAATAACGGTTTGGTTGATGCGAGTGATTTTGATGACGAGCGTAATGGCTGGCCTGTTGAACAAGTCTGGAAAGAAATGCATAAGCTTTTGCCATTCTCACCGGATTCAGTCGTCACTCATGGTGATTTCTCACTTGATAACCTTATTTTTGACGAGGGGAAATTAATAGGTTGTATTGATGTTGGACGAGTCGGAATCGCAGACCGATACCAGGATCTTGCCATCCTATGGAACTGCCTCGGTGAGTTTTCTCCTTCATTACAGAAACGGCTTTTTCAAAAATATGGTATTGATAATCCTGATATGAATAAATTGCAGTTTCATTTGATGCTCGATGAGTTTTTCTAA**TCAGTACTGACAATAAAAAGATTCTTGTTTTCAAGAACTTGTCATTTGTATAGTTTTTTTATATTGTAGTTGTTCTATTTTAATCAAATGTTAGCGTGATTTATATTTTTTTTCGCCTCGACATCATCTGCCCAGATGCGAAGTTAAGTGCGCAGAAAGTAATATCATGCGTCAATCGTATGTGAATGCTGGTCGCTATACTGCTGTCGATTCGATACTAACGCCGCCATCCAGTTGGTGCGTTTTGCGGAGGCTTAATTTTTTGAAGTTTATTTAATACTATCCTACATATGTACATTAAATACTTCCGTAACGTTTATCAATAAGAGTGGAAGATGCGCAATTATATTCAAAAGATTGGCCAGTCAATTAACTTAAGGAAAAAATTTACTGCGCGGCTGTGGTCCCATTTGAAACGGGGACGTTAGTGTTGCTTTTGTGTTTCCTTCTTCTTCTTTTTTTCTTTTCTTTAGAAGTATCTTCTTTGTCTTTCACCTCATCGCTGGGCGAGTTGACTTTCTTCTTCTTCTTTACGCTCAAAGGATTAGGAGCCTTTGGACCAAGCTTTCTTTTCTTAGTGATAGATTCCTTTCCTGATCCATCACCACTTTCTTGAAGTTTTTCAATATTAGGATCATTGAGACCCTTATACAATTTTTGTTCTTCAGTAATCTTGCTTGCCTTGGCACTCGCCGTACTGAGAGGTTCCATAACCATAACAGACCTGGTTAAATGGATTAATGGAACACCCGGAACCGTCCTCAGCTTCCTCCTCAAATCTATGTCCTGAGAAGCAACCACATACCTGTGTTTATTTGCACCGC

**(589 bp 3’ UTR to *DNL4* shown)**

**GFY-2383**

*prHIS3::****(u2)****::prGAL1/10::****SpCas9****::****NLS****::ADH1(t)****::****prMX****::Kan^R^::****MX(t)****::(u2)****::HIS3(t)*

***(u2) 20 bp target and 3 bp PAM sequence***

**(992 bp 5’ UTR to *HIS3* shown)**

GGGTCAGTTATTTCATCCAGATATAACCCGAGAGGAAACTTCTTAGCGTCTGTTTTCGTACCATAAGGCAGTTCATGAGGTATATTTTCGTTATTGAAGCCCAGCTCGTGAATGCTTAATGCTGCTGAACTGGTGTCCATGTCGCCTAGGTACGCAATCTCCACAGGCTGCAAAGGTTTTGTCTCAAGAGCAATGTTATTGTGCACCCCGTAATTGGTCAACAAGTTTAATCTGTGCTTGTCCACCAGCTCTGTCGTAACCTTCAGTTCATCGACTATCTGAAGAAATTTACTAGGAATAGTGCCATGGTACAGCAACCGAGAATGGCAATTTCTACTCGGGTTCAGCAACGCTGCATAAACGCTGTTGGTGCCGTAGACATATTCGAAGATAGGATTATCATTCATAAGTTTCAGAGCAATGTCCTTATTCTGGAACTTGGATTTATGGCTCTTTTGGTTTAATTTCGCCTGATTCTTGATCTCCTTTAGCTTCTCGACGTGGGCCTTTTTCTTGCCATATGGATCCGCTGCACGGTCCTGTTCCCTAGCATGTACGTGAGCGTATTTCCTTTTAAACCACGACGCTTTGTCTTCATTCAACGTTTCCCATTGTTTTTTTCTACTATTGCTTTGCTGTGGGAAAAACTTATCGAAAGATGACGACTTTTTCTTAATTCTCGTTTTAAGAGCTTGGTGAGCGCTAGGAGTCACTGCCAGGTATCGTTTGAACACGGCATTAGTCAGGGAAGTCATAACACAGTCCTTTCCCGCAATTTTCTTTTTCTATTACTCTTGGCCTCCTCTAGTACACTCTATATTTTTTTATGCCTCGGTAATGATTTTCATTTTTTTTTTTCCACCTAGCGGATGACTCTTTTTTTTTCTTAGCGATTGGCATTATCACATAATGAATTATACATTATATAAAGTAATGTGATTTCTTCGAAGAATATACTAAAAAATGAGCAGGCAAGATAAACGAAGGCAAAG**GCTGTTCGTGTGCGCGTCCTGGG**GACAGGTTATCAGCAACAACACAGTCATATCCATTCTCAATTAGCTCTACCACAGTGTGTGAACCAATGTATCCAGCACCACCTGTAACCAAAACAATTTTAGAAGTACTTTCACTTTGTAACTGAGCTGTCATTTATATTGAATTTTCAAAAATTCTTACTTTTTTTTTGGATGGACGCAAAGAAGTTTAATAATCATATTACATGGCATTACCACCATATACATATCCATATACATATCCATATCTAATCTTACTTATATGTTGTGGAAATGTAAAGAGCCCCATTATCTTAGCCTAAAAAAACCTTCTCTTTGGAACTTTCAGTAATACGCTTAACTGCTCATTGCTATATTGAAGTACGGATTAGAAGCCGCCGAGCGGGTGACAGCCCTCCGAAGGAAGACTCTCCTCCGTGCGTCCTCGTCTTCACCGGTCGCGTTCCTGAAACGCAGATGTGCCTCGCGCCGCACTGCTCCGAACAATAAAGATTCTACAATACTAGCTTTTATGGTTATGAAGAGGAAAAATTGGCAGTAACCTGGCCCCACAAACCTTCAAATGAACGAATCAAATTAACAACCATAGGATGATAATGCGATTAGTTTTTTAGCCTTATTTCTGGGGTAATTAATCAGCGAAGCGATGATTTTTGATCTATTAACAGATATATAAATGCAAAAACTGCATAACCACTTTAACTAATACTTTCAACATTTTCGGTTTGTATTACTTCTTATTCAAATGTAATAAAAGTATCAACAAAAAATTGTTAATATACCTCTATACTTTAACGTCAAGGAGAAAAAACTATA**ATGGATAAGAAATACTCTATCGGTTTGGATATTGGTACAAATTCAGTTGGTTGGGCAGTTATTACTGATGAATACAAGGTTCCATCTAAAAAGTTTAAAGTTTTGGGTAACACTGATAGACATTCTATTAAGAAAAATTTGATTGGTGCTTTGTTATTTGATTCTGGTGAAACTGCTGAAGCAACAAGATTGAAAAGAACTGCAAGAAGAAGATACACAAGAAGAAAGAATAGAATCTGTTATTTGCAAGAAATTTTCTCTAACGAAATGGCTAAGGTTGATGATTCTTTCTTTCATAGATTGGAAGAATCATTTTTAGTTGAAGAAGATAAGAAACATGAAAGACATCCAATCTTCGGTAACATCGTTGATGAAGTTGCTTACCATGAAAAGTACCCAACAATCTATCATTTGAGAAAGAAATTGGTTGATTCAACTGATAAGGCAGATTTGAGATTGATATATTTGGCTTTAGCACATATGATCAAGTTTAGAGGTCATTTCTTGATCGAGGGTGACTTGAATCCAGATAATTCTGATGTTGATAAGTTGTTTATTCAATTAGTTCAAACATATAATCAATTGTTTGAAGAAAATCCAATTAATGCTTCTGGTGTTGATGCTAAGGCAATCTTGTCAGCAAGATTGTCTAAGTCAAGAAGATTGGAAAATTTGATCGCTCAATTACCAGGTGAAAAGAAAAATGGTTTGTTCGGTAATTTGATCGCATTGTCTTTGGGTTTGACACCAAACTTCAAGTCAAACTTCGATTTGGCTGAAGATGCAAAGTTGCAATTGTCTAAGGATACTTACGATGATGATTTGGATAATTTGTTGGCTCAAATTGGTGACCAATATGCAGATTTGTTTTTGGCTGCTAAAAATTTGTCTGATGCTATCTTGTTGTCAGATATCTTGAGAGTTAACACTGAAATCACAAAGGCTCCATTGTCTGCATCAATGATCAAGAGATACGATGAACATCATCAAGATTTGACTTTGTTGAAGGCATTGGTTAGACAACAATTACCAGAAAAGTACAAGGAAATTTTCTTTGATCAATCTAAAAATGGTTATGCTGGTTACATTGATGGTGGTGCATCTCAAGAAGAATTCTACAAGTTTATTAAGCCAATCTTGGAAAAGATGGATGGTACAGAAGAATTGTTAGTTAAATTGAACAGAGAAGATTTGTTAAGAAAACAAAGAACTTTCGATAACGGTTCTATCCCACATCAAATCCATTTGGGTGAATTACATGCTATCTTGAGAAGACAAGAAGATTTCTACCCATTTTTAAAGGATAACAGAGAAAAGATTGAAAAGATTTTGACTTTTAGAATTCCATATTACGTTGGTCCATTAGCTCGTGGTAATTCTAGATTTGCATGGATGACTAGAAAGTCAGAAGAAACTATCACACCATGGAATTTTGAAGAAGTTGTTGATAAAGGTGCTTCTGCACAATCTTTTATTGAAAGAATGACAAACTTCGATAAAAATTTGCCAAACGAAAAGGTTTTGCCAAAGCATTCATTGTTATATGAATACTTTACTGTTTACAATGAATTGACAAAAGTTAAATATGTTACTGAGGGTATGAGAAAACCAGCATTTTTGTCTGGTGAACAAAAGAAAGCAATCGTTGATTTGTTGTTTAAAACTAACAGAAAGGTTACAGTTAAACAATTGAAAGAAGATTACTTTAAGAAAATTGAATGTTTTGATTCTGTTGAAATTTCAGGTGTTGAAGATAGATTCAATGCTTCATTAGGTACTTACCATGATTTGTTGAAGATTATTAAGGATAAAGATTTCTTGGATAATGAAGAAAATGAAGATATTTTAGAAGATATTGTTTTAACTTTGACATTATTTGAAGATAGAGAAATGATCGAAGAAAGATTGAAGACATACGCTCATTTGTTCGATGATAAAGTTATGAAGCAATTGAAGAGAAGAAGATACACTGGTTGGGGTAGATTGTCTAGAAAGTTGATTAATGGTATCAGAGATAAGCAATCTGGTAAAACAATCTTGGATTTCTTGAAGTCAGATGGTTTCGCAAACAGAAACTTCATGCAATTGATTCATGATGATTCATTGACTTTTAAAGAAGATATCCAAAAAGCTCAAGTTTCTGGTCAGGGTGACTCATTGCATGAACATATTGCTAATTTGGCAGGTTCTCCAGCTATTAAGAAAGGTATCTTGCAAACAGTTAAGGTTGTTGATGAATTAGTTAAAGTTATGGGTAGACATAAGCCAGAAAACATCGTTATCGAAATGGCTAGAGAAAACCAAACTACACAAAAGGGTCAAAAGAATTCAAGAGAAAGAATGAAGAGAATCGAAGAAGGTATTAAAGAATTGGGTTCTCAAATCTTGAAGGAACATCCAGTTGAAAACACTCAATTGCAAAACGAAAAGTTGTACTTATACTACTTACAAAACGGTAGAGATATGTACGTTGATCAAGAATTAGATATCAACAGATTGTCAGATTACGATGTTGATCATATCGTTCCACAATCATTTTTGAAGGATGATTCAATCGATAATAAGGTTTTGACAAGATCTGATAAGAACCGTGGTAAATCTGATAATGTTCCATCAGAAGAAGTTGTTAAGAAAATGAAGAACTACTGGAGACAATTGTTAAATGCTAAGTTGATCACTCAAAGAAAGTTCGATAATTTGACAAAAGCTGAAAGAGGTGGTTTGTCAGAATTAGATAAAGCAGGTTTTATTAAGAGACAATTAGTTGAAACTAGACAAATCACAAAGCATGTTGCACAAATCTTGGATTCTAGAATGAACACTAAATATGATGAAAATGATAAATTAATTAGAGAAGTTAAAGTTATTACATTAAAATCTAAATTGGTTTCAGATTTTAGAAAAGATTTTCAATTCTACAAAGTTAGAGAAATTAATAACTATCATCATGCTCATGATGCATACTTGAATGCTGTTGTTGGTACTGCATTGATTAAGAAATACCCAAAGTTGGAATCTGAATTCGTTTACGGTGACTACAAGGTTTACGATGTTAGAAAGATGATCGCTAAGTCAGAACAAGAAATCGGTAAAGCTACAGCAAAGTATTTCTTTTATTCTAACATCATGAATTTCTTTAAAACTGAAATTACATTAGCTAACGGTGAAATCAGAAAAAGACCATTGATCGAAACTAATGGTGAAACAGGTGAAATTGTTTGGGATAAAGGTAGAGATTTCGCAACTGTTAGAAAGGTTTTGTCAATGCCACAAGTTAACATCGTTAAGAAAACTGAAGTTCAAACAGGTGGTTTTTCTAAGGAATCAATCTTGCCAAAGAGAAACTCTGATAAGTTGATTGCTAGAAAGAAAGATTGGGATCCAAAGAAATATGGTGGTTTTGATTCTCCAACTGTTGCTTACTCAGTTTTAGTTGTTGCAAAGGTTGAAAAGGGTAAATCTAAGAAATTGAAATCAGTTAAAGAATTGTTAGGTATCACAATCATGGAAAGATCTTCATTCGAAAAGAATCCAATCGATTTCTTGGAAGCAAAGGGTTACAAGGAAGTTAAGAAAGATTTGATTATTAAGTTGCCAAAGTACTCTTTGTTCGAATTAGAAAACGGTAGAAAAAGAATGTTAGCTTCAGCTGGTGAATTGCAAAAGGGTAATGAATTGGCTTTGCCATCTAAGTACGTTAATTTCTTGTATTTGGCATCTCATTACGAAAAGTTGAAGGGTTCACCAGAAGATAATGAACAAAAACAATTGTTCGTTGAACAACATAAGCATTATTTGGATGAAATTATTGAACAAATTTCTGAATTTTCAAAAAGAGTTATTTTGGCTGATGCAAATTTGGATAAGGTTTTGTCTGCTTACAATAAGCATAGAGATAAGCCAATCAGAGAACAAGCAGAAAACATCATCCATTTGTTTACTTTGACAAATTTGGGTGCTCCAGCTGCTTTTAAATACTTCGATACTACAATCGATAGAAAAAGATACACTTCTACAAAGGAAGTTTTGGATGCAACATTGATCCATCAATCAATCACTGGTTTGTATGAAACAAGAATTGATTTGTCTCAATTGGGTGGTGACTCTAGGGCAGACCCAAAGAAAAAGAGGAAAGTATAA**GGCGCGCCACTTCTAAATAAGCGAATTTCTTATGATTTATGATTTTTATTATTAAATAAGTTATAAAAAAAATAAGTGTATACAAATTTTAAAGTGACTCTTAGGTTTTAAAACGAAAATTCTTATTCTTGAGTAACTCTTTCCTGTAGGTCAGGTTGCTTTCTCAGGTATAGTATGAGGTCGCTCTTATTGACCACACCTCTACCGGCAGATCCGCTAGGGATAACAGGGTAATAT**AGATCT**GTTTAGCTTGCCTCGTCCCCGCCGGGTCACCCGGCCAGCGACATGGAGGCCCAGAATACCCTCCTTGACAGTCTTGACGTGCGCAGCTCAGGGGCATGATGTGACTGTCGCCCGTACATTTAGCCCATACATCCCCATGTATAATCATTTGCATCCATACATTTTGATGGCCGCACGGCGCGAAGCAAAAATTACGGCTCCTCGCTGCAGACCTGCGAGCAGGGAAACGCTCCCCTCACAGACGCGTTGAATTGTCCCCACGCCGCGCCCCTGTAGAGAAATATAAAAGGTTAGGATTTGCCACTGAGGTTCTTCTTTCATATACTTCCTTTTAAAATCTTGCTAGGATACAGTTCTCACATCACATCCGAACATAAACAACC**ATGGGTAAGGAAAAGACTCACGTTTCGAGGCCGCGATTAAATTCCAACATGGATGCTGATTTATATGGGTATAAATGGGCTCGCGATAATGTCGGGCAATCAGGTGCGACAATCTATCGATTGTATGGGAAGCCCGATGCGCCAGAGTTGTTTCTGAAACATGGCAAAGGTAGCGTTGCCAATGATGTTACAGATGAGATGGTCAGACTAAACTGGCTGACGGAATTTATGCCTCTTCCGACCATCAAGCATTTTATCCGTACTCCTGATGATGCATGGTTACTCACCACTGCGATCCCCGGCAAAACAGCATTCCAGGTATTAGAAGAATATCCTGATTCAGGTGAAAATATTGTTGATGCGCTGGCAGTGTTCCTGCGCCGGTTGCATTCGATTCCTGTTTGTAATTGTCCTTTTAACAGCGATCGCGTATTTCGTCTCGCTCAGGCGCAATCACGAATGAATAACGGTTTGGTTGATGCGAGTGATTTTGATGACGAGCGTAATGGCTGGCCTGTTGAACAAGTCTGGAAAGAAATGCATAAGCTTTTGCCATTCTCACCGGATTCAGTCGTCACTCATGGTGATTTCTCACTTGATAACCTTATTTTTGACGAGGGGAAATTAATAGGTTGTATTGATGTTGGACGAGTCGGAATCGCAGACCGATACCAGGATCTTGCCATCCTATGGAACTGCCTCGGTGAGTTTTCTCCTTCATTACAGAAACGGCTTTTTCAAAAATATGGTATTGATAATCCTGATATGAATAAATTGCAGTTTCATTTGATGCTCGATGAGTTTTTCTAA**TCAGTACTGACAATAAAAAGATTCTTGTTTTCAAGAACTTGTCATTTGTATAGTTTTTTTATATTGTAGTTGTTCTATTTTAATCAAATGTTAGCGTGATTTATATTTTTTTTCGCCTCGACATCATCTGCCCAGATGCGAAGTTAAGTGCGCAGAAAGTAATATCATGCGTCAATCGTATGTGAATGCTGGTCGCTATACTGCTGTCGATTCGATACTAACGCCGCCATCCAGT**GCTGTTCGTGTGCGCGTCCTGGG**TGACACCGATTATTTAAAGCTGCAGCATACGATATATATACATGTGTATATATGTATACCTATGAATGTCAGTAAGTATGTATACGAACAGTATGATACTGAAGATGACAAGGTAATGCATCATTCTATACGTGTCATTCTGAACGAGGCGCGCTTTCCTTTTTTCTTTTTGCTTTTTCTTTTTTTTTCTCTTGAACTCGAGAAAAAAAATATAAAAGAGATGGAGGAACGGGAAAAAGTTAGTTGTGGTGATAGGTGGCAAGTGGTATTCCGTAAGAACAACAAGAAAAGCATTTCATATTATGGCTGAACTGAGCGAACAAGTGCAAAATTTAAGCATCAACGACAACAACGAGAATGGTTATGTTCCTCCTCACTTAAGAGGAAAACCAAGAAGTGCCAGAAATAACAGTAGCAACTACAATAACAACAACGGCGGCTACAACGGTGGCCGTGGCGGTGGCAGCTTCTTTAGCAACAACCGTCGTGGTGGTTACGGCAACGGTGGTTTCTTCGGTGGAAACAACGGTGGCAGCAGATCTAACGGCCGTTCTGGTGGTAGATGGATCGATGGCAAACATGTCCCAGCTCCAAGAAACGAAAAGGCCGAGATCGCCATATTTGGTGTCCCCGAGGATCCAAATTTCCAATCTTCTGGTATTAACTTCGATAACTACGATGATATTCCAGTGGACGCCTCTGGTAAGGATGTTCCTGAACCAATCACAGAATTTACCTCACCTCCATTGGACGGATTGTTATTGGAAAACATCAAATTGGCCCGTTTCACCAAGCCAACACCTGTGCAAAAATACTCCGTCCCTATCGTTGCCAACGGCAGAGATTTGATGGCCTGTGCGCAGACCGGTTCTGGTAAGACTGGTGGGTTTTTATTCCCAGTGTTGTCCGAATCATTTAAGACTGGACCATCTCCTCAACCAGAGTCTCAAGGCTCCTTTTACCAAAGAAAGGCCTACCCAACTGCTGTCATTA

**(993 bp 3’ UTR to *HIS3* shown)**

**GFY-3864**

*prDNL4****::DNL4(WT)::****DNL4(t)*

***DNL4 Cas9 Target sites are displayed along with the immediate reading frame. Silent mutations are illustrated.***

**(590 bp 5’ UTR to *DNL4* shown)**

GGATGATGGGTAACATAAATAGAAGGGGTAATGGTTCGCAATCTGACACATCAGAGAGTGAGGAAAACTCAGAACAATCTGATTTGGAAGGCAATAATCAATGTATTGAATATGACTCTTTAGGTAATGCTATTCGTATAGATAACATGAAAAGCAGGGAAGCGCAATCTGAGGAATCAGAAGACGAGGAAAGTGGTTCAAAAGAAAATGGAGAGCCTTTAAGTTATGACCCCTTAGGCAATTTAATTCGATAGGTGATTAAATAGGCTGAAATCAGTGTTTAGTAACTACGTACGTTGTACATGTAACATTGTGATATAAATCGTAAGATTCGCCGAGTATAGATCAATAATATCGGTTTCATCACTTACGTTGTTTGTGCAGTACTAGAGTTAAGATCGTTTTCGATCCCTTATTTTCTTCTTTTTTCCTTTTTTTTGTTATTTTTCTCTTTTTACCTTTTGTCACCATATTAAATCTTTAAACAAATCTAACTATGAAAAAATCCTTTAAACATATGTTAATATGTGGAAAATAAATACTAAAATAAAAATCTAGAACTGAAGGAAATAGTAACGGATTATTTAGGT**ATGATATCAGCACTAGATTCTATACCCGAGCCCCAAAACTTTGCGCCTAGTCCAGATTTCAAATGGCTTTGTGAAGAGCTATTTGTGAAGATACATGAAGTTCAAATTAATGGAACGGCCGGCACTGGCAAATCAAGGTCTTTCAAGTACTATGAAATAATATCGAATTTCGTCGAAATGTGGAGAAAAACCGTGGGAAATAATATATATCCTGCACTGGTTCTTGCTCTTCCCTACCGCGATAGACGAATCTATAATATTAAGGATTATGTATTAATAAGAACTATATGCTCTTACTTGAAGTTGCCAAAAAATTCTGCAACAGAGCAGCGGTTAAAAGATTGGAAACAGCGTGTCGGTAAAGGTGGGAATCTTTCTTCTCTTCTTGTGGAAGAAATTGCTAAAAGAAGGGCTGAACCTAGCTCAAAAGCGATTACAATTGATAACGTCAATCACTATCTGGATAGTTTGAGTGGAGACAGGTTCGCTTCCGGACGAGGATTTAAGAGTCTTGTCAAGTCCAAACCTTTCCTGCACTGTGTGGAGAATATGAGTTTCGTCGAATTAAAATACTTCTTTGATATCGTGCTTAAAAATAGAGTAATAGGAGGTCAAGAGCACAAATTGCTAAACTGCTGGCATCCTGATGCTCAGGATTATCTTAGCGTGATATCTGATTTAAAGGTGGTAACTTCAAAACTTTATGATCCAAAAGTTCGTCTAAAGGATGATGATTTGAGTATAAAAGTTGGCTTTGCATTCGCCCCCCAATTAGCCAAAAAAGTGAATCTTTCTTATGAGAAAATATGCCGTACACTACATGATGATTTTTTGGTAGAAGAAAAAATGGATGGAGAACGAATTCAAGTTCATTATATGAATTATGGTGAATCCATAAAATTTTTTAGTAGACGGGGCATCGACTATACCTATTTGTACGGAGCGAGCTTATCATCAGGAACTATATCTCAACATTTGAGGTTTACAGATAGTGTTAAAGAATGTGTTTTAGATGGAGAAATGGTGACGTTTGATGCAAAAAGACGGGTGATTCTTCCATTCGGTCTTGTTAAAGGAAGTGCAAAGGAAGCGCTATCTTTTAATAGTATAAATAATGTTGACTTTCACCCCTTATATATGGTGTTTGATCTGTTATACCTGAATGGGACTTCGTTGACACCATTACCCCTTCATCAAAGGAAGCAATATCTGAACAGCATTTTAAGTCCCTTGAAAAATATTGTAGAAATAGTACGATCTTCTAGATGTTATGGTGTGGAGTCAATCAAAAAGTCTTTAGAAGTTGCAATCTCACTGGGTTCAGAAGGAGTTGTTTTGAAATATTATAATTCAAGTTATAATGTCGCCAGTCGAAACAACAACTGGATCAAGGTAAAACCTGAATATTTGGAGGAATTTGGAGAGAATTTAGACTTAATAGTAATAGGCAGAGATTCTGGGAAAAAAGATTCTTTTATGCTAGGGTTACTTGTGCTAGATGAAGAAGAGTATAAAAAGCACCAAGGAGACTCCTCTGAAATTGTAGACCACTCAAGCCAAGAAAAACACATACAAAATTCAAGAAGAAGGGTGAAAAAAATACTTTCATTCTGTTCTATCGCAAACGGTATATCTCAAGAAGAATTCAAAGAAATCGACCGCAAAACGAGAGGACATTGGAAAAGAACCTCCGAAGTTGCTCCCCCTGCTTCAATTTTAGAATTTGGCTCAAAAATACCTGCCGAATGGATTGACCCCAGTGAATCAATTGTTCTAGAAATAAAATCACGGTCTTTGGATAACACAGAAACGAATATGCAGAAGTACGCTACCAATTGTACTTTGTACGGTGGCTATTGTAAAAGAATACGGTACGATAAAGAATGGACAGATTGTTACACACTTAACGACTTATACGAAAGTAGGACGGTTAAATCTAACCCCAGCTATCAAGCGGAAAGGTCACAGCTTGGATTGATACGGAAAAAGAGAAAGAGAGTACTTATTTCAGACAGCTTTCACCAAAACAGGAAACAACTGCCAATTTCAAACATCTTTGCCGGATTACTTTTTTATGTTCTCTCTGACTATGTCACTGAAGATACTGGAATACGGATTACACGGGCAGAACTTGAAAAAACTATTGTGGAACATGGTGGTAAACTGATATATAATGTAATTTTAAAACGTCATTCAATTGGGGACGTTCGGTTAATCAGCTGTAAAACTACCACGGAATGCAAGGCTTTAATAGATCGAGGATATGATATATTGCACCCAAATTGGGTACTCGATTGTATAGCATATAAGAGGCTCATCCTGATCGAGCCCAATTATTGCTTTAACGTCTCTCAAAAAATGAGAGCCGTCGCTGAAAAAAGGGTAGATTGTTTGGGTGATAGTTTTGAAAATGACATTTCGGAAACCAAACTGTCATCATTGTATAAATCACAACTAAGTCTACCACCGATGGGGGAACTCGAGATAGATTCTGAGGTTCGGCGGTTTCCATTATTTTTATTCTCCAACAGGATTGCATACGTACCACGTCGCAAAATTAGCACAGAAGATGACATTATAGAAATGAAAATTAAGTTGTTTGGTGGAAAAATAACAGATCAACAGTCACTTTGTAACTTAATAATTATACCATATACTGATCCTATTTTGAGGAAAGACTGCATGAATGAGGTACACGAAAAAATAAAAGAACAAATAAAGGCTTCTGATACTATACCGAAAATAGCCAGGGTCGTTGCCCCTGAATGGGTGGATCATTCTATTAATGAAAACTGTCAAGTGCCTGAGGAGGATTTCCCCGTAGTCAACTACTGA**TGGTGCGTTTTGCGGAGGCTTAATTTTTTGAAGTTTATTTAATACTATCCTACATATGTACATTAAATACTTCCGTAACGTTTATCAATAAGAGTGGAAGATGCGCAATTATATTCAAAAGATTGGCCAGTCAATTAACTTAAGGAAAAAATTTACTGCGCGGCTGTGGTCCCATTTGAAACGGGGACGTTAGTGTTGCTTTTGTGTTTCCTTCTTCTTCTTTTTTTCTTTTCTTTAGAAGTATCTTCTTTGTCTTTCACCTCATCGCTGGGCGAGTTGACTTTCTTCTTCTTCTTTACGCTCAAAGGATTAGGAGCCTTTGGACCAAGCTTTCTTTTCTTAGTGATAGATTCCTTTCCTGATCCATCACCACTTTCTTGAAGTTTTTCAATATTAGGATCATTGAGACCCTTATACAATTTTTGTTCTTCAGTAATCTTGCTTGCCTTGGCACTCGCCGTACTGAGAGGTTCCATAACCATAACAGACCTGGTTAAATGGATTAATGGAACACCCGGAACCGTCCTCAGCTTCCTCCTCAAATCTATGTCCTGAGAAGCAACCACATACCTGTGTTTATTTGCACCGC

**(589 bp 3’ UTR to *DNL4* shown)**

**GFY-3875**

*prDNL4****::DNL4(WT)::CDC11(t)::sgRNA(Kan)::****DNL4(t)*

*prSNR52****::crRNA::****tracrRNA****::SUP4(t)***

**(590 bp 5’ UTR to *DNL4* shown)**

GGATGATGGGTAACATAAATAGAAGGGGTAATGGTTCGCAATCTGACACATCAGAGAGTGAGGAAAACTCAGAACAATCTGATTTGGAAGGCAATAATCAATGTATTGAATATGACTCTTTAGGTAATGCTATTCGTATAGATAACATGAAAAGCAGGGAAGCGCAATCTGAGGAATCAGAAGACGAGGAAAGTGGTTCAAAAGAAAATGGAGAGCCTTTAAGTTATGACCCCTTAGGCAATTTAATTCGATAGGTGATTAAATAGGCTGAAATCAGTGTTTAGTAACTACGTACGTTGTACATGTAACATTGTGATATAAATCGTAAGATTCGCCGAGTATAGATCAATAATATCGGTTTCATCACTTACGTTGTTTGTGCAGTACTAGAGTTAAGATCGTTTTCGATCCCTTATTTTCTTCTTTTTTCCTTTTTTTTGTTATTTTTCTCTTTTTACCTTTTGTCACCATATTAAATCTTTAAACAAATCTAACTATGAAAAAATCCTTTAAACATATGTTAATATGTGGAAAATAAATACTAAAATAAAAATCTAGAACTGAAGGAAATAGTAACGGATTATTTAGGT**ATGATATCAGCACTAGATTCTATACCCGAGCCCCAAAACTTTGCGCCTAGTCCAGATTTCAAATGGCTTTGTGAAGAGCTATTTGTGAAGATACATGAAGTTCAAATTAATGGAACGGCCGGCACTGGCAAATCAAGGTCTTTCAAGTACTATGAAATAATATCGAATTTCGTCGAAATGTGGAGAAAAACCGTGGGAAATAATATATATCCTGCACTGGTTCTTGCTCTTCCCTACCGCGATAGACGAATCTATAATATTAAGGATTATGTATTAATAAGAACTATATGCTCTTACTTGAAGTTGCCAAAAAATTCTGCAACAGAGCAGCGGTTAAAAGATTGGAAACAGCGTGTCGGTAAAGGTGGGAATCTTTCTTCTCTTCTTGTGGAAGAAATTGCTAAAAGAAGGGCTGAACCTAGCTCAAAAGCGATTACAATTGATAACGTCAATCACTATCTGGATAGTTTGAGTGGAGACAGGTTCGCTTCCGGACGAGGATTTAAGAGTCTTGTCAAGTCCAAACCTTTCCTGCACTGTGTGGAGAATATGAGTTTCGTCGAATTAAAATACTTCTTTGATATCGTGCTTAAAAATAGAGTAATAGGAGGTCAAGAGCACAAATTGCTAAACTGCTGGCATCCTGATGCTCAGGATTATCTTAGCGTGATATCTGATTTAAAGGTGGTAACTTCAAAACTTTATGATCCAAAAGTTCGTCTAAAGGATGATGATTTGAGTATAAAAGTTGGCTTTGCATTCGCCCCCCAATTAGCCAAAAAAGTGAATCTTTCTTATGAGAAAATATGCCGTACACTACATGATGATTTTTTGGTAGAAGAAAAAATGGATGGAGAACGAATTCAAGTTCATTATATGAATTATGGTGAATCCATAAAATTTTTTAGTAGACGGGGCATCGACTATACCTATTTGTACGGAGCGAGCTTATCATCAGGAACTATATCTCAACATTTGAGGTTTACAGATAGTGTTAAAGAATGTGTTTTAGATGGAGAAATGGTGACGTTTGATGCAAAAAGACGGGTGATTCTTCCATTCGGTCTTGTTAAAGGAAGTGCAAAGGAAGCGCTATCTTTTAATAGTATAAATAATGTTGACTTTCACCCCTTATATATGGTGTTTGATCTGTTATACCTGAATGGGACTTCGTTGACACCATTACCCCTTCATCAAAGGAAGCAATATCTGAACAGCATTTTAAGTCCCTTGAAAAATATTGTAGAAATAGTACGATCTTCTAGATGTTATGGTGTGGAGTCAATCAAAAAGTCTTTAGAAGTTGCAATCTCACTGGGTTCAGAAGGAGTTGTTTTGAAATATTATAATTCAAGTTATAATGTCGCCAGTCGAAACAACAACTGGATCAAGGTAAAACCTGAATATTTGGAGGAATTTGGAGAGAATTTAGACTTAATAGTAATAGGCAGAGATTCTGGGAAAAAAGATTCTTTTATGCTAGGGTTACTTGTGCTAGATGAAGAAGAGTATAAAAAGCACCAAGGAGACTCCTCTGAAATTGTAGACCACTCAAGCCAAGAAAAACACATACAAAATTCAAGAAGAAGGGTGAAAAAAATACTTTCATTCTGTTCTATCGCAAACGGTATATCTCAAGAAGAATTCAAAGAAATCGACCGCAAAACGAGAGGACATTGGAAAAGAACCTCCGAAGTTGCTCCCCCTGCTTCAATTTTAGAATTTGGCTCAAAAATACCTGCCGAATGGATTGACCCCAGTGAATCAATTGTTCTAGAAATAAAATCACGGTCTTTGGATAACACAGAAACGAATATGCAGAAGTACGCTACCAATTGTACTTTGTACGGTGGCTATTGTAAAAGAATACGGTACGATAAAGAATGGACAGATTGTTACACACTTAACGACTTATACGAAAGTAGGACGGTTAAATCTAACCCCAGCTATCAAGCGGAAAGGTCACAGCTTGGATTGATACGGAAAAAGAGAAAGAGAGTACTTATTTCAGACAGCTTTCACCAAAACAGGAAACAACTGCCAATTTCAAACATCTTTGCCGGATTACTTTTTTATGTTCTCTCTGACTATGTCACTGAAGATACTGGAATACGGATTACACGGGCAGAACTTGAAAAAACTATTGTGGAACATGGTGGTAAACTGATATATAATGTAATTTTAAAACGTCATTCAATTGGGGACGTTCGGTTAATCAGCTGTAAAACTACCACGGAATGCAAGGCTTTAATAGATCGAGGATATGATATATTGCACCCAAATTGGGTACTCGATTGTATAGCATATAAGAGGCTCATCCTGATCGAGCCCAATTATTGCTTTAACGTCTCTCAAAAAATGAGAGCCGTCGCTGAAAAAAGGGTAGATTGTTTGGGTGATAGTTTTGAAAATGACATTTCGGAAACCAAACTGTCATCATTGTATAAATCACAACTAAGTCTACCACCGATGGGGGAACTCGAGATAGATTCTGAGGTTCGGCGGTTTCCATTATTTTTATTCTCCAACAGGATTGCATACGTACCACGTCGCAAAATTAGCACAGAAGATGACATTATAGAAATGAAAATTAAGTTGTTTGGTGGAAAAATAACAGATCAACAGTCACTTTGTAACTTAATAATTATACCATATACTGATCCTATTTTGAGGAAAGACTGCATGAATGAGGTACACGAAAAAATAAAAGAACAAATAAAGGCTTCTGATACTATACCGAAAATAGCCAGGGTCGTTGCCCCTGAATGGGTGGATCATTCTATTAATGAAAACTGTCAAGTGCCTGAGGAGGATTTCCCCGTAGTCAACTACTGA**GTCCGCTTTTGGCTTCCTCACTTATTTCTTCTTTCTCTATATATATAAAGAGTGAGTGTTGTATATAAGTAAATACATCTGGTATATTATTTTTTTTTTTTCTTCATTCTTAAAAAGTATTAATATCGATCAGCAAAAAAAAATTAACAAAAAAGTTCCTTATTATATCTGCGTAGAAGTACTTATTTCTGCTCCACCTTTGGAGTATTTTTCCAAAATTGTGATGCCAAATGAGTAATGAAATAGAATTTCTTGTGTGGATCGTCATTATCGATTTGGTTTGGACAGTTAAAGGAGAATTTTGGAAAGACCAGAACATCACTTAGC**GGATCC**TCACTAAAGGGAACAAAAGCTGGAGCTTCTTTGAAAAGATAATGTATGATTATGCTTTCACTCATATTTATACAGAAACTTGATGTTTTCTTTCGAGTATATACAAGGTGATTACATGTACGTTTGAAGTACAACTCTAGATTTTGTAGTGCCCTCTTGGGCTAGCGGTAAAGGTGCGCATTTTTTCACACCCTACAATGTTCTGTTCAAAAGATTTTGGTCAAACGCTGTAGAAGTGAAAGTTGGTGCGCATGTTTCGGCGTTCGAAACTTCTCCGCAGTGAAAGATAAATGATC**GCCATCCTATGGAACTGCCT**GTTTTAGAGCTAGAAATAGCAAGTTAAAATAAGGCTAGTCCGTTATCAACTTGAAAAAGTGGCACCGAGTCGGTGGTGC**TTTTTTTGTTTTTTATGTCT**TCGAGTCATGTAATTAGTTATGTCACGC**CTCGAG**TGGTGCGTTTTGCGGAGGCTTAATTTTTTGAAGTTTATTTAATACTATCCTACATATGTACATTAAATACTTCCGTAACGTTTATCAATAAGAGTGGAAGATGCGCAATTATATTCAAAAGATTGGCCAGTCAATTAACTTAAGGAAAAAATTTACTGCGCGGCTGTGGTCCCATTTGAAACGGGGACGTTAGTGTTGCTTTTGTGTTTCCTTCTTCTTCTTTTTTTCTTTTCTTTAGAAGTATCTTCTTTGTCTTTCACCTCATCGCTGGGCGAGTTGACTTTCTTCTTCTTCTTTACGCTCAAAGGATTAGGAGCCTTTGGACCAAGCTTTCTTTTCTTAGTGATAGATTCCTTTCCTGATCCATCACCACTTTCTTGAAGTTTTTCAATATTAGGATCATTGAGACCCTTATACAATTTTTGTTCTTCAGTAATCTTGCTTGCCTTGGCACTCGCCGTACTGAGAGGTTCCATAACCATAACAGACCTGGTTAAATGGATTAATGGAACACCCGGAACCGTCCTCAGCTTCCTCCTCAAATCTATGTCCTGAGAAGCAACCACATACCTGTGTTTATTTGCACCGC

**(589 bp 3’ UTR to *DNL4* shown)**

**GFY-3856**

**The *HIS3* locus is identical to GFY-2383.**

*prDNL4****::****prMX****::SpHIS5::****MX(t)****::****DNL4(t)*

[Additional sequence from original yeast genome deletion strain library]

**(590 bp 5’ UTR to *DNL4* shown)**

GGATGATGGGTAACATAAATAGAAGGGGTAATGGTTCGCAATCTGACACATCAGAGAGTGAGGAAAACTCAGAACAATCTGATTTGGAAGGCAATAATCAATGTATTGAATATGACTCTTTAGGTAATGCTATTCGTATAGATAACATGAAAAGCAGGGAAGCGCAATCTGAGGAATCAGAAGACGAGGAAAGTGGTTCAAAAGAAAATGGAGAGCCTTTAAGTTATGACCCCTTAGGCAATTTAATTCGATAGGTGATTAAATAGGCTGAAATCAGTGTTTAGTAACTACGTACGTTGTACATGTAACATTGTGATATAAATCGTAAGATTCGCCGAGTATAGATCAATAATATCGGTTTCATCACTTACGTTGTTTGTGCAGTACTAGAGTTAAGATCGTTTTCGATCCCTTATTTTCTTCTTTTTTCCTTTTTTTTGTTATTTTTCTCTTTTTACCTTTTGTCACCATATTAAATCTTTAAACAAATCTAACTATGAAAAAATCCTTTAAACATATGTTAATATGTGGAAAATAAATACTAAAATAAAAATCTAGAACTGAAGGAAATAGTAACGGATTATTTAGGTATGGATGTCCACGAGGTCTCTCGCACGATTACAACATCTGACGTACGCTGCAGGTCGACGGATCCCCGGGTTAATTAAGGCGCGCCAGATCTGTTTAGCTTGCCTCGTCCCCGCCGGGTCACCCGGCCAGCGACATGGAGGCCCAGAATACCCTCCTTGACAGTCTTGACGTGCGCAGCTCAGGGGCATGATGTGACTGTCGCCCGTACATTTAGCCCATACATCCCCATGTATAATCATTTGCATCCATACATTTTGATGGCCGCACGGCGCGAAGCAAAAATTACGGCTCCTCGCTGCAGACCTGCGAGCAGGGAAACGCTCCCCTCACAGACGCGTTGAATTGTCCCCACGCCGCGCCCCTGTAGAGAAATATAAAAGGTTAGGATTTGCCACTGAGGTTCTTCTTTCATATACTTCCTTTTAAAATCTTGCTAGGATACAGTTCTCACATCACATCCGAACATAAACAACC**ATGGGTAGGAGGGCTTTTGTAGAAAGAAATACGAACGAAACGAAAATCAGCGTTGCCATCGCTTTGGACAAAGCTCCCTTACCTGAAGAGTCGAATTTTATTGATGAACTTATAACTTCCAAGCATACAAACCAAAAGGGAGAACAAGTAATCCAAGTAGACACGGGAATTGGATTCTTGGATCACATGTATCATGCACTGGCTAAACATGCAGGCTGGAGCTTACGACTTTACTCAAGAGGTGATTTAATCATCGATGATCATCACACTGCAGAAGATACTGCTATTGCACTTGGTATTGCATTCAAGCAGGCTATGAGTAACTTTGCCGGCGTTAAAAGATTTGGACATGCTTATTGTCCACTTGACGAAGCTCTTTCTAGAAGCGTAGTTGACTTGTCGGGACGGCCCTATGCTGTTATCGATTTGGGATTAAAGCGTGAAAAGGTTGGGGAATTGTCCTGTGAAATGATCCCTCACTTACTATATTCCTTTTCGGTAGCAGCTGGAATTACTTTGCATGTTACCTGCTTATATGGTAGTAATGACCATCATCGTGCTGAAAGCGCTTTTAAATCTCTGGCTGTTGCCATGCGCGCGGCTACTAGTCTTACTGGAAGTTCTGAAGTCCCAAGCACGAAGGGAGTGTTGTAA**AGAGTACTGACAATAAAAAGATTCTTGTTTTCAAGAACTTGTCATTTGTATAGTTTTTTTATATTGTAGTTGTTCTATTTTAATCAAATGTTAGCGTGATTTATATTTTTTTTCGCCTCGACATCATCTGCCCAGATGCGAAGTTAAGTGCGCAGAAAGTAATATCATGCGTCAATCGTATGTGAATGCTGGTCGCTATACTGCTGTCGATTCGATACTAACGCCGCCATCCAGTGTCGAAAACAAGCTCGAATTCATCGATTGTCTGATTGTGCGTGACCCCTACGAGACCGACACCGTGATGGTGCGTTTTGCGGAGGCTTAATTTTTTGAAGTTTATTTAATACTATCCTACATATGTACATTAAATACTTCCGTAACGTTTATCAATAAGAGTGGAAGATGCGCAATTATATTCAAAAGATTGGCCAGTCAATTAACTTAAGGAAAAAATTTACTGCGCGGCTGTGGTCCCATTTGAAACGGGGACGTTAGTGTTGCTTTTGTGTTTCCTTCTTCTTCTTTTTTTCTTTTCTTTAGAAGTATCTTCTTTGTCTTTCACCTCATCGCTGGGCGAGTTGACTTTCTTCTTCTTCTTTACGCTCAAAGGATTAGGAGCCTTTGGACCAAGCTTTCTTTTCTTAGTGATAGATTCCTTTCCTGATCCATCACCACTTTCTTGAAGTTTTTCAATATTAGGATCATTGAGACCCTTATACAATTTTTGTTCTTCAGTAATCTTGCTTGCCTTGGCACTCGCCGTACTGAGAGGTTCCATAACCATAACAGACCTGGTTAAATGGATTAATGGAACACCCGGAACCGTCCTCAGCTTCCTCCTCAAATCTATGTCCTGAGAAGCAACCACATACCTGTGTTTATTTGCACCGC

**(589 bp 3’ UTR to *DNL4* shown)**

**pGF-V809**

**pRS425 + sgRNA(u2)**

*prSNR52****::crRNA::****tracrRNA****::SUP4(t)***

**GGATCC**tcactaaagggaacaaaagctggagcttctttgaaaagataatgtatgattatgctttcactcatatttatacagaaacttgatgttttctttcgagtatatacaaggtgattacatgtacgtttgaagtacaactctagattttgtagtgccctcttgggctagcggtaaaggtgcgcattttttcacaccctacaatgttctgttcaaaagattttggtcaaacgctgtagaagtgaaagttggtgcgcatgtttcggcgttcgaaacttctccgcagtgaaagataaatgatc**GCTGTTCGTGTGCGCGTCCT**GTTTTAGAGCTAGAAATAGCAAGTTAAAATAAGGCTAGTCCGTTATCAACTTGAAAAAGTGGCACCGAGTCGGTGGTGC**TTTTTTTGTTTTTTATGTCT**tcgagtcatgtaattagttatgtcacgc**CTCGAG**

**pGF-V1220**

**pRS425 + sgRNA(u1)**

*prSNR52****::crRNA::****tracrRNA****::SUP4(t)***

**GGATCC**tcactaaagggaacaaaagctggagcttctttgaaaagataatgtatgattatgctttcactcatatttatacagaaacttgatgttttctttcgagtatatacaaggtgattacatgtacgtttgaagtacaactctagattttgtagtgccctcttgggctagcggtaaaggtgcgcattttttcacaccctacaatgttctgttcaaaagattttggtcaaacgctgtagaagtgaaagttggtgcgcatgtttcggcgttcgaaacttctccgcagtgaaagataaatgatc**CGGTGGACTTCGGCTACGTA**GTTTTAGAGCTAGAAATAGCAAGTTAAAATAAGGCTAGTCCGTTATCAACTTGAAAAAGTGGCACCGAGTCGGTGGTGC**TTTTTTTGTTTTTTATGTCT**tcgagtcatgtaattagttatgtcacgc**CTCGAG**

**Figure S3.** Analysis of haploid genomes of the triple drive and triple target yeast strains by diagnostic PCRs. Purified chromosomal preparations of GFY-3596 (targets) and GFY-3675 (drives) haploid yeast strains were analyzed by PCR at each locus (Targets 1-3). Agarose (1%) gels were imaged (*left*) with equal loads of PCR samples. Two images (*top*, unedited) of the same DNA gel are presented. Oligonucleotides used can be found in Supplemental Table S5 and the primers correspond to the gene drive system presented in Fig. 2A. The expected fragments sizes are illustrated (*right*). For the chosen PCRs at each target locus, identical reactions using the haploid triple drive genome as a template does not result in amplification of the expected fragment. All PCR reactions were performed using identical conditions.

**Figure S4.** Re-activation of gene drive system in clonal isolates displaying incomplete initial drive activity. Two isolates (13,14) from the [triple drive x triple target] cross displayed imperfect drive activity by both growth and diagnostic PCR (Fig. 2F). Both clonal isolates were confirmed as diploids as previously described^1^. Isolate-13 had maintained the sgRNA(u1) plasmid (marked with *LEU2*), and had activated Drive 1 at the *HIS3* locus (sensitivity on SD-HIS). Isolate-14 had lost the sgRNA(u1) plasmid when re-tested on SD-LEU medium; the plasmid was transformed back into this strain for a second round of activation. As in Fig. 2B, Cas9 expression was induced by culturing in galactose for 5 hr followed by plating onto SD-LEU medium for 2 days. Next, yeast were transferred by replica plating to SD-HIS, G418, and SD-URA medium and incubated for an additional 24 hr prior to imaging. The percentage of yeast colonies sensitive to each growth condition is displayed (red text).

**
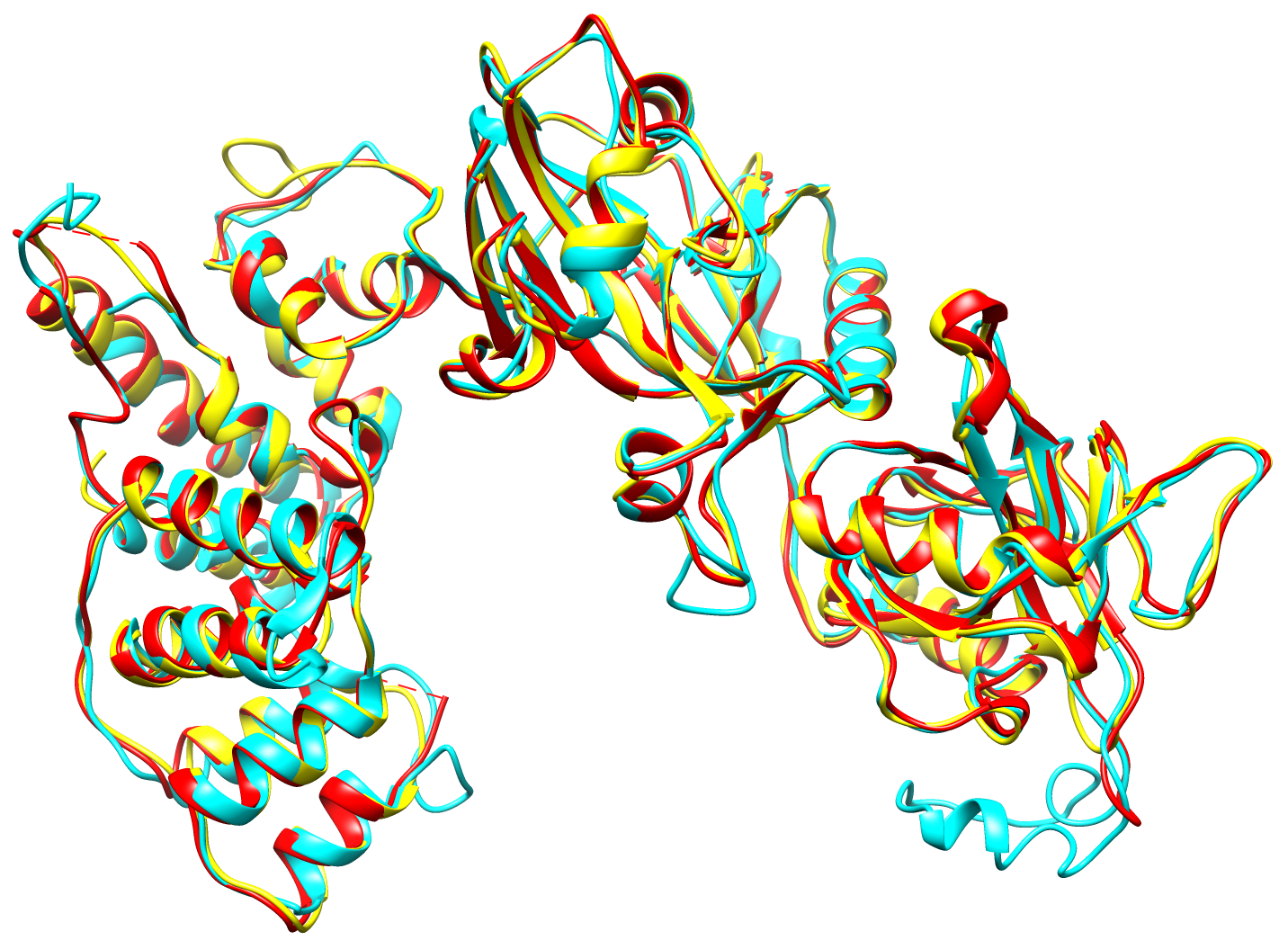
**

**Figure S5.** Conservation of the N-terminal domain of DNA Ligase IV. The primary sequences of the *S. cerevisiae* Dnl4 (1-648, teal), and *A. gambiae* Ligase IV (1-591, yellow) were modeled against the crystal structure of the N-terminus of human Lig4 (1-605, red) (PDB:3W1B) using I-TASSER^2^ and aligned using MatchMaker in Chimera^3^. Each I-TASSER model was individually aligned against the human Lig4 N-terminal structure.

**Figure S6.** Further analysis of clonal isolates from the triple gene drive harboring *dnl4* replacement alleles. Clonal isolates (2 per genotype) were obtained from the gene drive analysis from Fig. 4F. All yeast were re-tested on each media type for growth and ploidy status was confirmed as diploid (*below*). The WT control (GFY-3875) was mated to the triple target strain (GFY-3596), diploids were selected (three consecutive rounds), and chromosomal DNA was isolated from clonal samples (no galactose or raffinose/sucrose treatment). For all other drives (1-12), a 5 hr galactose induction was used (Fig. 4F). Diagnostic PCRs were performed on isolated genomic DNA for each locus for samples before (0 hr) and after (5 hr) drive activation. For the *DNL4* locus, one PCR (C) was used to examine the *dnl4::CDC11(t)::sgRNA(Kan)* construct whereas two PCRs (F and G) were used to assay for the presence or absence of the *dnl4∆::Kan^R^* target cassette. The oligonucleotides used can be found in Supplemental Table S5. These data illustrate that the three target loci have been lost following gene drive activation. Images of DNA bands were cropped from larger gels or from independent gels (separated by white lines). The unedited gel images can be found in Supplemental Fig. S9.

**Figure S7.** Original images of yeast agar plates used within this study. The two sets of plates used in Fig. 2B are included, unedited. Plates were scanned and not processed further.

**Figure S8.** Original images of DNA agarose gels used for PCRs from Fig. 2F and Fig. 4D are included with molecular markers. (A) PCRs from the 0 hr time point (2 isolates) are included on unedited gels. The red asterisk designates PCR lanes not included within the final figures. (B) PCRs from the 5 hr time point (12 isolates) are included on unedited gels. (C) A gel containing the PCRs for isolates 13 and 14 from Fig. 2F. (D) PCRs from Fig. 4D are included on three unedited DNA gels. Isolates (A-P) are included on two gels whereas isolates (Q-F’) are included on a third gel. All gel images shown were collected from the Invitrogen E-Gel^TM^ Imager (ThermoFisher Scientific), cropped for clarity, but were not processed by any method.

**Figure S9.** Original DNA gels from Supplemental Fig. S6. Molecular markers are included. PCRs from both 0 hr and 5 hr time points are included. Red asterisk, PCR lanes not included in the final figure. All gel images shown were collected as in Fig. S8.

**Table S1.** Yeast strains used in this study.

| **Strain** | **Genotype** | **Reference** |
| --- | --- | --- |
| BY4741 | *MAT***a** *his3Δ1 leu2Δ0 met15Δ0 ura3Δ0* | 4 |
| BY4742 | *MAT⍺ his3Δ1 leu2Δ0 lys2Δ0 ura3Δ0* | 4 |
| GFY-3675^a^ | BY4741; *his3∆::prHIS3:(u2)::prGAL1/10::SpCas9::NLS::ADH1(t):(u2)::HIS3(t) shs1∆::prSHS1::sgRNA(GFP)::SHS1(t) dnl4∆::prDNL4::sgRNA(Kan)::DNL4(t)* | This study |
| GFY-3206^b^ | BY4742; *his3∆::prHIS3::(u1)::prCDC12::mCherry::NLS::SHS1(t)::prCCW12::SpHIS5::*  *MX(t)::(u1)::HIS3(t)* | 1 |
| GFY-3593^c^ | BY4742; *shs1∆::prSHS1::SHS1::GFP::CDC10(t)::prMX::CaURA3::SHS1(t)* | This study |
| GFY-3264b^d^ | BY4742; *dnl4∆::prDNL4::prMX::Kan^R^::MX(t)::DNL4(t)* | 4 |
| GFY-3578 | BY4742; *his3∆::prHIS3::(u1)::prCDC12::mCherry::NLS::SHS1(t)::prCCW12::SpHIS5::*  *MX(t)::(u1)::HIS3(t)*  *shs1∆::prSHS1::SHS1::GFP::CDC10(t)::prMX::CaURA3::SHS1(t)* | This study |
| GFY-3594 | BY4742; *his3∆::prHIS3::(u1)::prCDC12::mCherry::NLS::SHS1(t)::prCCW12::SpHIS5::*  *MX(t)::(u1)::HIS3(t)*  *dnl4∆::prDNL4::prMX::Kan^R^::MX(t)::DNL4(t)* | This study |
| GFY-3623 | BY4742;  *shs1∆::prSHS1::SHS1::GFP::CDC10(t)::prMX::CaURA3::SHS1(t)*  *dnl4∆::prDNL4::prMX::Kan^R^::MX(t)::DNL4(t)* | This study |
| GFY-3596^e^ | BY4742; *his3∆::prHIS3::(u1)::prCDC12::mCherry::NLS::SHS1(t)::prCCW12::SpHIS5::*  *MX(t)::(u1)::HIS3(t)*  *shs1∆::prSHS1::SHS1::GFP::CDC10(t)::prMX::CaURA3::SHS1(t)*  *dnl4∆::prDNL4::prMX::Kan^R^::MX(t)::DNL4(t)* | This study |
| GFY-3611 | BY4741; *his3∆::prHIS3:(u2)::prGAL1/10::SpCas9::NLS::ADH1(t):(u2)::HIS3(t) shs1∆::prSHS1::sgRNA(GFP)::SHS1(t) DNL4* | This study |
| GFY-2383 | BY4741; *his3∆::prHIS3:(u2)::prGAL1/10::SpCas9::NLS::ADH1(t):prMX::*  *Kan^R^::MX(t)::(u2)::HIS3(t)* | 1 |
| GFY-3850^f^ | BY4741; *his3∆::prHIS3:(u2)::prGAL1/10::SpCas9::NLS::ADH1(t):prMX::*  *Kan^R^::MX(t)::(u2)::HIS3(t) dnl4∆::prDNL4::dnl4(K742A)::DNL4(t)* | This study |
| GFY-3851 | BY4741; *his3∆::prHIS3:(u2)::prGAL1/10::SpCas9::NLS::ADH1(t):prMX::*  *Kan^R^::MX(t)::(u2)::HIS3(t) dnl4∆::prDNL4::dnl4(T744A)::DNL4(t)* | This study |
| GFY-3852 | BY4741; *his3∆::prHIS3:(u2)::prGAL1/10::SpCas9::NLS::ADH1(t):prMX::*  *Kan^R^::MX(t)::(u2)::HIS3(t) dnl4∆::prDNL4::dnl4(K742A T744A)::DNL4(t)* | This study |
| GFY-3853 | BY4741; *his3∆::prHIS3:(u2)::prGAL1/10::SpCas9::NLS::ADH1(t):prMX::*  *Kan^R^::MX(t)::(u2)::HIS3(t) dnl4∆::prDNL4::dnl4(L750::STOP)::DNL4(t)* | This study |
| GFY-3854 | BY4741; *his3∆::prHIS3:(u2)::prGAL1/10::SpCas9::NLS::ADH1(t):prMX::*  *Kan^R^::MX(t)::(u2)::HIS3(t) dnl4∆::prDNL4::dnl4(D800K)::DNL4(t)* | This study |
| GFY-3855 | BY4741; *his3∆::prHIS3:(u2)::prGAL1/10::SpCas9::NLS::ADH1(t):prMX::*  *Kan^R^::MX(t)::(u2)::HIS3(t) dnl4∆::prDNL4::dnl4(G868A G869A)::DNL4(t)* | This study |
| GFY-3864 | BY4741; *his3∆::prHIS3:(u2)::prGAL1/10::SpCas9::NLS::ADH1(t):prMX::*  *Kan^R^::MX(t)::(u2)::HIS3(t) dnl4∆::prDNL4::DNL4(WT)::DNL4(t)* | This study |
| GFY-3856^g^ | BY4741; *his3∆::prHIS3:(u2)::prGAL1/10::SpCas9::NLS::ADH1(t):prMX::*  *Kan^R^::MX(t)::(u2)::HIS3(t) dnl4∆::prDNL4::prMX::SpHIS5::MX(t)::DNL4(t)* | This study |
| GFY-3865^h^ | BY4741; *his3∆::prHIS3:(u2)::prGAL1/10::SpCas9::NLS::ADH1(t):(u2)::HIS3(t) shs1∆::prSHS1::sgRNA(GFP)::SHS1(t) dnl4∆::prDNL4::dnl4(K742A)::CDC11(t)::sgRNA(Kan)::DNL4(t)* | This study |
| GFY-3866 | BY4741; *his3∆::prHIS3:(u2)::prGAL1/10::SpCas9::NLS::ADH1(t):(u2)::HIS3(t) shs1∆::prSHS1::sgRNA(GFP)::SHS1(t) dnl4∆::prDNL4::dnl4(T744A)::CDC11(t)::sgRNA(Kan)::DNL4(t)* | This study |
| GFY-3867 | BY4741; *his3∆::prHIS3:(u2)::prGAL1/10::SpCas9::NLS::ADH1(t):(u2)::HIS3(t) shs1∆::prSHS1::sgRNA(GFP)::SHS1(t) dnl4∆::prDNL4::dnl4(K742A T744A)::CDC11(t)::sgRNA(Kan)::DNL4(t)* | This study |
| GFY-3871 | BY4741; *his3∆::prHIS3:(u2)::prGAL1/10::SpCas9::NLS::ADH1(t):(u2)::HIS3(t) shs1∆::prSHS1::sgRNA(GFP)::SHS1(t) dnl4∆::prDNL4::dnl4(D800K)::CDC11(t)::sgRNA(Kan)::DNL4(t)* | This study |
| GFY-3872 | BY4741; *his3∆::prHIS3:(u2)::prGAL1/10::SpCas9::NLS::ADH1(t):(u2)::HIS3(t) shs1∆::prSHS1::sgRNA(GFP)::SHS1(t) dnl4∆::prDNL4::dnl4(G868A G869A)::CDC11(t)::sgRNA(Kan)::DNL4(t)* | This study |
| GFY-3875 | BY4741; *his3∆::prHIS3:(u2)::prGAL1/10::SpCas9::NLS::ADH1(t):(u2)::HIS3(t) shs1∆::prSHS1::sgRNA(GFP)::SHS1(t) dnl4∆::prDNL4::DNL4(WT)::CDC11(t)::sgRNA(Kan)::DNL4(t)* | This study |

^a^Strain GFY-3675 was derived from GFY-2383. First, *S. pyogenes* Cas9 expression was activated as previously described^1^ followed by transformation of the sgRNA(Kan) plasmid (pGF-V1642) and an amplified PCR fragment: *ADH1(t)::(u2)::HIS3(t)* which included 168 bp of the *ADH1* terminator, the 23 bp unique (u2) sequence (5’ GCTGTTCGTGTGCGCGTCCT**GGG** 3’) where the PAM is in bold^5^, and 263 bp of the *HIS3* terminator. Yeast were plated on SD-LEU medium. DSB formation followed by genomic repair via HR removed the Kan^R^ cassette entirely. Editing was followed by strain propagation on rich media (dextrose) to inhibit Cas9 expression and allow for loss of the high-copy sgRNA-containing plasmid. Second, *SHS1* was deleted using an amplified knock-out cassette (from pGF-V170) and 500 bp of flanking UTR. Next, Cas9-based editing was performed using the sgRNA(Kan) plasmid and repair DNA: *prSHS1::sgRNA(GFP)::SHS1(t)*. Third, *DNL4* was deleted using the knock-out cassette from strain GFY-3264b. Cas9 editing allowed for integration of *prDNL4::sgRNA(Kan)::DNL4(t)* (amplified from pGF-IVL1498) at the native locus. All strains and intermediates were confirmed by diagnostic PCR and DNA sequencing of all manipulated loci. The final strain was propagated for multiple weeks ensuring complete loss of both residual Cas9 and high copy plasmid used for construction.

^b^The unique (u1) sequence (5’ ATGACGGTGGACTTCGGCTACGTA**GGG**CGATT 3’) includes a PAM (bold) and the 20 bp target site for *Sp*Cas9 (underlined)^5^. The *HIS5* gene (functional equivalent to *S. cerevisiae* *HIS3*) is from *Schizosaccharomyces pombe.*

^c^Strain GFY-3593 was constructed by direct transformation and integration of a PCR fragment: *prSHS1::SHS1::GFP::CDC10(t)::prMX::CaURA3::SHS1(t)*. The *Candida albicans* *URA3* gene (from plasmid JT-2868) does not include the standard *MX(t)* sequence and, instead, uses the native *SHS1* 3’ UTR.

^d^The haploid (MAT*⍺*) *dnl4∆::Kan^R^* strain was confirmed using multiple diagnostic PCR. The isogenic isolate was from the yeast haploid genome deletion collection (UC Berkeley).

^e^Strain GFY-3596 was derived from GFY-3206 and required multiple rounds of HR-based integration at the *SHS1* and *DNL4* loci.

^f^Strains GFY-3850 to 3855, and GFY-3864 were constructed by first generating a plasmid construct containing the last 1559 bp of the *DNL4* coding sequence and 589 bp of the *DNL4* 3’ UTR sequence. Consecutive rounds of a modified PCR mutagenesis protocol^6^ introduced 6 silent polymorphisms into the *DNL4* gene; these occur within codons 699, 700, 701, 936, 937 and 938. These were designed within two separate native Cas9 target sites (labeled as “A” and “B”) within *DNL4*: (5’ GACTATGTCACTGAAGATAC**TGG** 3’) and (5’ **CCT**GAGGAGGATTTCCCCGTAGT 3’) where the PAM (bold) and target sequences (underlined) are marked. Additional mutation(s) were then added to *DNL4.* Second, strain GFY-2383 was induced for Cas9 expression and transformed with two separate high-copy plasmids (pYY-DNL4(A) and pYY-DNL4(B), marked with *LEU2/URA3*, respectively) to target *DNL4* at the dual targets (A and B) as well as repair DNA (594 bp of upstream homology of the cleavage site (A) and 589 bp of downstream homology within the terminator) PCR amplified from pYY-IVL6 to pYY-IVL10, pYY-IVL12, and pYY-IVL14 to yield the seven yeast strains. Viable yeast were selected on SD-LEU-URA medium and confirmed for subsequent loss of both sgRNA-containing plasmids by growth on rich medium.

^g^Strain GFY-3856 was generated by first switching the selectable marker in GFY-3264b from Kan^R^ to *SpHIS5*. Second, the entire *dnl4∆::SpHIS5* locus was PCR amplified and transformed into GFY-2383.

^h^Strains GFY-3865 to GFY-3867, GFY-3871, GFY-3872, and GFY-3875 were generated using a similar methodology to GFY-3850 with several modifications. First, Cas9-based editing was performed in GFY-3611 as the parent strain. Second, the integrating construct included the *DNL4* coding sequence followed by 327 bp of the *CDC11* terminator, the 455 bp sgRNA(Kan) expression construct, and 589 bp of the *DNL4* 3’ UTR. Third, donor PCRs were amplified from pYY-IVL1 to pYY-IVL3, pYY-IVL5, pYY-IVL11, and pYY-IVL13 to generate appropriate strains.

**Table S2**. Plasmids used in this study.

| **Plasmid** | **Description** | **Reference** |
| --- | --- | --- |
| pRS425 | 2μ, *LEU2* | 7 |
| pRS426 | 2μ, *URA3* | 7 |
| pGF-V1220^a^ | pRS425; *prSNR52::Sp-sgRNA(u1)::SUP4(t)* | 1 |
| pGF-V809^b^ | pRS425; *prSNR52::Sp-sgRNA(u2)::SUP4(t)* | 1 |
| pYY-DNL4(A)^c^ | pRS425; *prSNR52::Sp-sgRNA(u2)::SUP4(t)* | This study |
| pYY-DNL4(B)^d^ | pRS426; *prSNR52::Sp-sgRNA(u2)::SUP4(t)* | This study |

^a^The sgRNA(u1) sequence is 5’ CGGTGGACTTCGGCTACGTA 3’^5^. All sgRNA constructs include 269 bp of the *SNR52* promoter, the 79 bp tracrRNA, and the 20 bp *SUP4* terminator, as modeled from^8^.

^b^The sgRNA(u1) sequence is 5’ GCTGTTCGTGTGCGCGTCCT 3’.

^c^The sgRNA(DNL4-A) sequence is 5’ GACTATGTCACGGAGGACAC 3’.

^d^The sgRNA(DNL4-B) sequence is 5’ ACTACGGGGAAGTCTTCTTC 3’. The target is present on the non-coding strand at the 3’ end of the *DNL4* gene.

**Table S3**. Species used for alignments and phylogeny of DNA Ligase IV.

| **Species Name (Strain) [Protein Name]** | **Amino Acids** | **Reference ID** |
| --- | --- | --- |
| *Saccharomyces cerevisiae* (S288C) [Dnl4] | 944 | NCBI Ref Seq: NP_014647.1 |
| *Candida glabrata* [DNA ligase 4] | 946 | GenBank: KTB12236.1 |
| *Candida albicans* (P75016) [DNA ligase 4] | 928 | GenBank: KHC72222.1 |
| *Schizosaccharomyces* *pombe* [Lig4] | 913 | NCBI Ref Seq: NP_587888.2 |
| *Neurospora* *crassa* (OR74A) [Mus53] | 1050 | NCBI Ref Seq: XP_962868.2 |
| *Yarrowia* *lipolytica* [hypothetical protein YALI1_D27044g] | 956 | GenBank: AOW04412.1 |
| *Papilio* *machaon* [DNA ligase 4, predicted] | 880 | NCBI Ref Seq: XP_014356497.1 |
| *Drosophila* *melanogaster* [Ligase4] | 918 | NCBI Ref Seq: NP_572907.1 |
| *Anopheles* *gambiae* (str. PEST) [AGAP000623-PA] | 914 | NCBI Ref Seq: XP_310455.4 |
| *Aedes* *aegypti* [AAEL017365-PA, partial] | 925 | GenBank: EJY57630.1 |
| *Zootermopsis* *nevadensis* [DNA ligase 4] | 899 | NCBI Ref Seq: XP_021923135.1 |
| *Danio* *rerio* [DNA ligase 4] | 909 | NCBI Ref Seq: NP_001096593.1 |
| *Xenopus* *laevis* [DNA ligase IV] | 911 | GenBank: AAL56554.1 |
| *Gallus* *gallus* [DNA ligase IV] | 912 | GenBank: BAB68506.1 |
| *Mus* *musculus* [DNA ligase 4] | 911 | NCBI Ref Seq: NP_795927.2 |
| *Rattus* *norvegicus* [DNA ligase 4] | 911 | NCBI Ref Seq: NP_001099565.1 |
| *Homo* *sapiens* [DNA ligase 4 isoform 1] | 911 | NCBI Ref Seq: NP_002303.2 |
| *Bos taurus* [DNA ligase 4] | 911 | NCBI Ref Seq: NP_001178055.1 |

**Table S4**. Accuracy metrics for I-TASSER modeling of DNA Ligase IV structure.

**I-TASSER Template:**

Human Lig4 NT (1-605) (PDB:3W1B):

| **I-TASSER Models** | **C-score**^a^ | **TM-score**^b^ | **RMSD**^c^ |
| --- | --- | --- | --- |
| Yeast Dnl4 NT (1-648) | 0.51 | 0.78 ± 0.10 | 8.8 ± 4.0Å |
| Mosquito Lig4 NT (1-591) | 1.92 | 0.99 ± 0.04 | 3.7 ± 2.5Å |

**I-TASSER Template:**

Yeast Dnl4 CT (683-939) (PDB:1Z56):

| **I-TASSER Models** | **C-score** | **TM-score** | **RMSD** |
| --- | --- | --- | --- |
| Human Lig4 CT (656-911) | 0.27 | 0.75 ± 0.10 | 5.3 ± 3.4Å |
| Mosquito Lig4 CT (645-914) | 0.10 | 0.73 ± 0.11 | 5.8 ± 3.6Å |

^a^The C-score is a metric for the confidence of the quality of the predicted I-TASSER model. This score usually ranges from -5 to 2 (higher value, higher confidence)^2^.

^b^The TM-score is a metric for structural similarity and scores > 0.5 indicate high confidence ^9^.

^c^Root-mean-square deviation of atomic positions.

**Table S5.** Oligonucleotides used for diagnostic PCRs.

| **Oligonucleotide Name** | **DNA Sequence (5’ to 3’)** |
| --- | --- |
| F1: *prHIS3* +196 F | GGCCTCCTCTAGTACACTCTATATTTTTTTATGC |
| R1: Int *S.p.Cas9* +373 R | CATCAACGATGTTACCGAAGATTGGATGTC |
| F2: *prSHS1* +497 F | GTTCCTCCGGTTTAAACTGATCCATAGTGATAG |
| R2: *SHS1(t)* -192 R | GCCATATTTAAATTTATCCCTACAATTATTTGACACTGTTTG |
| F3: *prDNL4* +590 F | GGATGATGGGTAACATAAATAGAAGGGGTAATG |
| R3: sgRNA clone out R1 | CTCGAGGCGTGACATAACTAATTACATGACTC |
| F4: Int *SpHIS5* F1 | GGGAGAACAAGTAATCCAAGTAGACACGGG |
| R4: *HIS3(term)* -151 R | CGCCTCGTTCAGAATGACACGTATAGAATG |
| F5: Int GFP F new | GGTGTTCAATGCTTTTCAAGATACCCAGATC |
| R5: *CDC10(term)* -201 R | CAAACGAGAAGGTGATAGCTGTACGCCAG |
| R6: Internal Kan R | GAACACTGCCAGCGCATCAACAATATTTTC |
| F6: *prHIS3* +500 F | TTCTCGACGTGGGCCTTTTTCTTGCCATAT |
| F7: Internal Kan F | CGGTTGCATTCGATTCCTGTTTGTAATTGTCC |
| R7: *HIS3(term)* -497 R | GTAACCACCACGACGGTTGTTGCTAAAGAA |
| F8: Int *DNL4* +1308 F | GTGTGGAGTCAATCAAAAAGTCTTTAGAAGTTGCA |
| F9: *prDNL4* +666 F | GCTGAAGAGGAATGGTTGCAAAAATTAGAAAAGG |
| R8: MX clone out R2 | ACTGGATGGCGGCGTTAGTATCGAATCGA |

**REFERENCES**

1 Roggenkamp, E. *et al.* Tuning CRISPR-Cas9 Gene Drives in Saccharomyces cerevisiae. *G3 (Bethesda, Md.)* **8**, 999-1018, doi:10.1534/g3.117.300557 (2018).

2 Roy, A., Kucukural, A. & Zhang, Y. I-TASSER: a unified platform for automated protein structure and function prediction. *Nature protocols* **5**, 725-738, doi:10.1038/nprot.2010.5 (2010).

3 Pettersen, E. F. *et al.* UCSF Chimera--a visualization system for exploratory research and analysis. *Journal of computational chemistry* **25**, 1605-1612, doi:10.1002/jcc.20084 (2004).

4 Brachmann, C. B. *et al.* Designer deletion strains derived from Saccharomyces cerevisiae S288C: a useful set of strains and plasmids for PCR-mediated gene disruption and other applications. *Yeast (Chichester, England)* **14**, 115-132, doi:10.1002/(sici)1097-0061(19980130)14:2<115::aid-yea204>3.0.co;2-2 (1998).

5 Finnigan, G. C. & Thorner, J. mCAL: a new approach for versatile multiplex action of Cas9 using one sgRNA and loci flanked by a programmed target sequence. *G3 (Bethesda, Md.)* **6**, 2147-2156, doi:10.1534/g3.116.029801 (2016).

6 Zheng, L., Baumann, U. & Reymond, J. L. An efficient one-step site-directed and site-saturation mutagenesis protocol. *Nucleic acids research* **32**, e115, doi:10.1093/nar/gnh110 (2004).

7 Christianson, T. W., Sikorski, R. S., Dante, M., Shero, J. H. & Hieter, P. Multifunctional yeast high-copy-number shuttle vectors. *Gene* **110**, 119-122 (1992).

8 DiCarlo, J. E. *et al.* Genome engineering in Saccharomyces cerevisiae using CRISPR-Cas systems. *Nucleic acids research* **41**, 4336-4343, doi:10.1093/nar/gkt135 (2013).

9 Zhang, Y. & Skolnick, J. Scoring function for automated assessment of protein structure template quality. *Proteins* **57**, 702-710, doi:10.1002/prot.20264 (2004).
